# Energetic costs of cellular and therapeutic control of stochastic mitochondrial DNA populations

**DOI:** 10.1371/journal.pcbi.1007023

**Published:** 2019-06-26

**Authors:** Hanne Hoitzing, Payam A. Gammage, Lindsey Van Haute, Michal Minczuk, Iain G. Johnston, Nick S. Jones

**Affiliations:** 1 Department of Mathematics, Imperial College London, London, SW7 2AZ, United Kingdom; 2 MRC Mitochondrial Biology Unit, University of Cambridge, Cambridge, CB2 0XY, United Kingdom; 3 CRUK Beatson Institute for Cancer Research, Glasgow, United Kingdom; 4 Faculty of Mathematics and Natural Sciences, University of Bergen, Bergen, Norway; 5 Alan Turing Institute, London, United Kingdom; University of Michigan, UNITED STATES

## Abstract

The dynamics of the cellular proportion of mutant mtDNA molecules is crucial for mitochondrial diseases. Cellular populations of mitochondria are under homeostatic control, but the details of the control mechanisms involved remain elusive. Here, we use stochastic modelling to derive general results for the impact of cellular control on mtDNA populations, the cost to the cell of different mtDNA states, and the optimisation of therapeutic control of mtDNA populations. This formalism yields a wealth of biological results, including that an increasing mtDNA variance can increase the energetic cost of maintaining a tissue, that intermediate levels of heteroplasmy can be more detrimental than homoplasmy even for a dysfunctional mutant, that heteroplasmy distribution (not mean alone) is crucial for the success of gene therapies, and that long-term rather than short intense gene therapies are more likely to beneficially impact mtDNA populations.

## Author summary

Mitochondria, best known for their role in energy production, are crucial to the survival of most of our cells. To respond to energetic demands and mitigate against mutational damage, cells control the mitochondrial populations within them. However, the character of these control mechanisms remains open. As experimental elucidation of these mechanisms is challenging, theoretical approaches can help us understand the general principles of cellular control of mitochondria in physiology and disease. Here, we use stochastic modelling to compare control strategies by studying their impact on the dynamics of mitochondrial DNA (mtDNA) populations as well as their energetic burden to the cell. We identify optimal strategies for the cell to control against mtDNA damage and preserve energy production and use this theory to explore the action of recently developed mitochondrial gene therapies, which reduce the fraction of mutant mtDNA molecules inside cells. We show how treatment efficiency may depend on pre-treatment distributions of mutant and wildtype mtDNA molecules: treatments are less effective for tissues consisting of cells with highly varying mutant levels, and long-term, rather than short intense, gene therapies should be favoured.

## Introduction

Most human cells contain 100-10,000 copies of mitochondrial DNA (mtDNA) which are situated inside the mitochondria. The proteins encoded by mtDNA are crucial for mitochondrial functionality, and mutations in mtDNA can cause devastating diseases [1–6]. Heteroplasmy, the proportion of mutant mtDNA molecules in a cell, typically has to pass a certain threshold (∼ 60-95%) before any biochemical defects can be observed [7–14]. The existence of thresholds at which mutant loads begin to have an effect has profound implications for our understanding of disease onset, drawing attention to the variance dynamics of the mutant fraction in cellular populations. As this variance increases more cells can be above threshold, and thus show pathology, even if average mutant load is unchanged.

Mitochondrial biogenesis and maintenance require cellular resources, and mitochondria are key sources of ATP and play other important metabolic roles. The particular ‘effective cost’ that cellular control of mitochondria acts to minimise remains poorly understood: for example, both decreases [15] and increases [15, 16] in wildtype copy numbers have been observed for different mutations as the mutant load increases. Some studies suggest that mtDNA density is controlled [17–19], others that total mtDNA mass [20, 21], or mtDNA transcription rate [22] is controlled. Understanding mtDNA population dynamics inside cells, and how these populations react to clinical interventions, is crucial in understanding diseases [23, 24]. However, experimental tracking of mtDNA populations over time is challenging, necessitating predictive mathematical modelling to provide a quantitative understanding.

In parallel with efforts to elucidate cell physiological control, protein engineering methods to artificially control mtDNA heteroplasmy are making fast progress. Two recently developed methods for cleaving DNA at specific sites involve zinc finger nucleases (ZFNs) and transcription activator-like effector nucleases (TALENs) [25–31], which have been re-engineered to specifically cleave mutant mtDNA [32–36]. MitoTALENs have been successfully used to reduce mutant loads in cells containing disease-related mutations, but elimination of the target mutant mtDNA was not complete [32, 37]. Similarly, treating cells multiple times with mtZFNs led to near-complete elimination of mutant mtDNAs [35, 36]. Quantitative theory for these therapeutic technologies has not yet been developed, leaving open questions about how these tools can be optimally deployed.

In this paper, we develop theory from bottom-up bioenergetic principles which allows us to study the effects of distinct cellular mtDNA control strategies, to analyse the bioenergetic cost of different mtDNA states, and to combine mtDNA control and energy-based cost to identify optimal control strategies for the cell. Finally, we construct a model for therapeutic mtDNA control using recent experimental data [36] and highlight challenges linked to heteroplasmy variance.

## Results

### Control: General insights on the role of feedback control

We employ a linear form of mtDNA feedback control and assume each mtDNA molecule replicates and degrades according to Poisson processes with rates λ and *μ*, respectively. Because control of biogenesis or autophagy yield similar behaviours [38], we assume that the degradation rate *μ* is constant and that feedback control is manifest through the replication rate λ(*w*, *m*), where *w* and *m* denote the number of mutant and wildtype mtDNA molecules in the cell. To connect with experiments, we use *μ* ≈ 0.07 day^−1^ corresponding to a half-life of about 10 days [39]. We only model post-mitotic cells, though our analysis can be extended to include cell divisions.

Specifically, we use a birth rate of the form:

$$\begin{array}{c} \lambda \left(w,m\right)=\mu +c_{1}\left(w_{opt}-\left(w+\delta m\right)\right) \end{array}$$ (1)

where *c*_1_ > 0, *w*_*opt*_ > 0 and *δ* are constants, with *w*_*opt*_ denoting the steady state value towards which the effective population, here defined as *w* + *δm*, is controlled. The magnitude of *c*_1_ determines how tightly the population is controlled. We use the term ‘mitochondrial sensing’ to describe how the cell might sense the mitochondrial population that is present. ‘Mutant sensing’ then refers to how strongly mutants are sensed relatively to wildtypes, which is encoded in the parameter *δ*. When steady state is reached (i.e. *w* + *δm* = *w*_*opt*_), replication and degradation rates are equal. In the absence of mutants, the resulting wildtype steady state is assumed to be optimal. We note that assuming the existence of *w*_*opt*_ does not imply a control based on copy number. Other quantities related to mitochondria may be controlled instead, such as total mitochondrial mass or ATP production, their desired values being reached at an effective population size of *w*_*opt*_. Thus, we define ‘mitochondrial sensing’ to refer to a wide range of mechanisms available to the cell to infer properties of its mitochondrial population, which can then be used to decide on a control action.

The deterministic dynamics resulting from this control are described in Eq (4). We do not include the possibility of *de novo* mutations but our approach can straightforwardly describe the subsequent behaviour if new mutations arise. Our linear model shares features with the ‘relaxed replication model’ [40, 41] (Eq (5)), though is written in a simpler form. The relaxed replication model has been used in a variety of other models [42, 43] and has obtained experimental support [15].

We will first investigate properties of more general control strategies, after which we return to our linear control and discuss parameterisations that optimise the energy status of the cell. Finally, we use the linear control to fit recent experimental data involving treatment of heteroplasmic cells with mtZFNs.

#### A wide range of control strategies induces similar mtDNA behaviour and admits quantitative analysis

**Fig 1:**
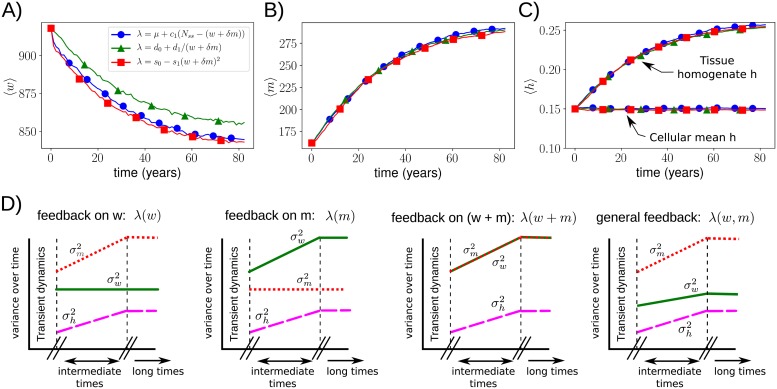
**A-C: A wide range of cellular control strategies can yield similar dynamics**. Stochastic simulations were used to compare three structurally distinct cellular controls (see legend), each reflecting a different function of the underlying sensed quantity *w* + *δm* with *δ* = 0.5. All controls are set to have the same wildtype mean and variance in the absence of mutants (section 2 in S1 File). No explicit selection for either mtDNA species is used. Figure (C) illustrates the difference between cellular mean and tissue homogenate heteroplasmy. **D: Control tradeoffs are required when multiple species are present**. The more strongly one species is controlled, the more control is lost over the other. Changes in variances between cells as described by the linear noise approximation (section 1 in S1 File) are shown (intermediate times). For long times, fixation occurs and the variance of the surviving species saturates. In the most-right figure we depicted the case in which mutants contribute less to the control than wildtypes (*δ* < 1).

Many possible control strategies can be parameterised to give rise to nearly identical means and variances for wildtype, mutant, and heteroplasmy dynamics up to long times (e.g. a human life-time) (Fig 1A, 1B and 1C, S1B, S1C and S1D Fig). This is especially true when mtDNA copy numbers fluctuate around their steady state values, in which case a linear control forms a good first order approximation to the complex ‘true’ control function. In this case, it is not the manner in which the controlled quantity is being controlled, but **which** quantity is controlled that is the most important. For example, the extent to which mutants and wildtypes contribute to the replication feedback function (determined by *δ*) determines how their relative means and variances evolve (Fig 1D), and contains more information on the dynamics than the functional form of λ(*w*, *m*) for fixed *δ* (e.g. whether λ(*w*, *m*) is linear or quadratic).

We stress the difference between two types of average heteroplasmy, as was also stressed in Ref. [41]: the individual cellular mean heteroplasmy ${\langle h\rangle }_{cellular}=\frac{1}{n_{\text{cells}}}\sum_{\text{cells}\;\text{i}}\frac{m_{i}}{m_{i}+w_{i}}$ (with *n*_*cells*_ the number of cells in the tissue) and the tissue homogenate heteroplasmy ${\langle h\rangle }_{homog}=\left(\sum_{\text{cells}\;\text{i}}m_{i}\right)/\left(\sum_{\text{cells}\;\text{i}}\left(m_{i}+w_{i}\right)\right)$. This difference is clearly seen in Fig 1C. When no explicit selection is present for either mtDNA species, mean cellular heteroplasmy remains constant at its initial value *m*_0_/(*m*_0_ + *w*_0_), where *w*_0_ and *m*_0_ denote the initial wildtype and mutant copy numbers, respectively. The homogenate heteroplasmy at long times is given by *m*_0_/(*m*_0_ + *δw*_0_) (section 2.2 in S1 File). It is clear that when mutants contribute little to the feedback control (small *δ*), tissue homogenate heteroplasmy can reach high values, and even approach 〈*h*〉_*homog*_ = 1, without explicit selection. A tissue can thus appear, when studying the homogenate heteroplasmy, to show selection for one type of mtDNA over another, whereas in fact mean cellular heteroplasmy is unaltered and mutant and wildtypes have identical proliferation rates.

#### Nonlinear cost functions predict changes in tissue maintenance

The birth-death model used to describe mtDNA dynamics can be written as a master equation (Methods, section 1 in S1 File). S1 Table shows the first order solution of the system size expansion, an approximation method to master equations, which is known as the linear noise approximation (LNA). Applying this approximation to a general form of mtDNA control (section 1 in S1 File), we find that i) if only one species is controlled, the variance of this species quickly reaches a constant value (see also [38]), ii) when both species are controlled with equal strength their variances increase at identical rates, iii) in general the more tightly controlled species has a more slowly increasing variance, and iv) the rate of increase of heteroplasmy variance depends, to first order, only on mtDNA copy number and turnover (as found in [38]) (Fig 1, Table 1(I)). Eventually, all variances reach a constant value due to fixation.

**Table 1:** Key results.

	Key results presented in this paper.
I	If only one mtDNA species is controlled the variance of the controlled species reaches a constant value. When both species are controlled with equal strength their variances increase at identical rates, and, in general, the more tightly controlled species has a more slowly increasing variance (Fig 1). [D]
II	The mean energetic cost of maintaining a tissue can increase over time due to the nonlinear influence of mtDNA variance, even if the energetic demand on the tissue stays the same and mean levels of mtDNA are constant (Eq 6). [D]
III	Intermediate heteroplasmy states can be more expensive than states homoplasmic in either mutant or wildtype. [C]
IV	A control lacking any mutant contribution can show an exponentially increasing cost, and the effects of particular cellular control strategies are more pronounced in low copy number cells (Fig 3A and 3B). [D, C]
V	Control strategies based on the energy status of the cell can often outperform control based on mtDNA copy number or sensing mtDNA mass (which would work well for deficient deletion mutants, but would be suboptimal for deficient point mutations) (Fig 3C). [D,C]
VI	Even for pathological mutants, reduction of mutant mtDNA alone is not always the optimal control strategy for a cell to adopt (Fig 4). [C]
VII	Tissues with high mean heteroplasmy levels will generally be harder to treat with mitochondrially targeted endonucleases if the heteroplasmy variance is high, especially if this high mean level is caused by a small percentage of cells (Fig 6A and 6B). [D,T]
VIII	Weak long-term rather than short intense endonuclease treatments are more likely to beneficially impact mtDNA populations (Fig 6D and 6E). [D,T]

Here we present key results of this paper, which hold under the assumptions used in our models (see text and Discussion). We place in square brackets the models we invoke for each part: D—our model for mitochondrial dynamics; C—a particular illustrative family of cost functions; T—a model for gene therapy.

What are the biological implications of these findings? A given mtDNA state (*w*, *m*) will accrue a cost to the cell, denoted by *C*(*w*, *m*), which can e.g. be an energetic cost or some other metric of tissue burden. If this cost function is nonlinear, increasing variances in *w* and *m* can lead to changes in mean cost 〈*C*(*w*, *m*)〉 even when mean cellular copy numbers 〈*w*〉, 〈*m*〉 remain constant (Methods) because the mean of a nonlinear function of random variables is not generally equal to the function of the mean of those variables (as seen above with cellular vs homogenate heteroplasmy). Therefore, the mean cost of maintaining a tissue may increase over time, even if tissue demands and mean mtDNA levels stay constant (Table 1(II)). However, these increases may be small and their significance depends on the details of the cost function: hence the need to consider explicit forms, as we do in the next section.

### Cost: An effective mitochondrial energy-based cost function

Next, to find general quantitative principles underlying mitochondrial energy budgets, we build a cost function that assigns a cost to any given mtDNA state (*w*, *m*) and allows a general quantitative investigation of the tradeoffs in maintaining cellular mtDNA populations. The ‘true’ energy budget of a cell with a given mitochondrial population is highly complex, involving many different metabolic processes in which mitochondria are involved [44–46]. We provide a simpler description, focussing on ATP production as a central mitochondrial function, and removing kinetic details in favour of a coarse-grained representation, to provide qualitative rather than quantitative results.

#### General cost function structure

Three important terms involved in the energy status of a cell are: i) the energy demand *D*, ii) the net energy supply *S*, and iii) the efficiency of energy supply. Here we define efficiency as the amount of energy produced per unit of resource consumed. We included intuitive and general terms in our energy-based cost function, such as replication, degradation and maintenance costs, supply and demand, and resource availability. We seek a cost function that captures the idea that there might be an optimal number of mitochondria: not so few that each mitochondrion is inefficiently overworked and not so many that the burden of the mitochondrial population is itself large.

We express our effective cost function as:

$$\begin{array}{c} C\left(w,m\right)=|D-S\left(w,m\right)|+\alpha \left(wr_{w}+mr_{m}\right) \end{array}$$ (2)

where *α* is a scaling constant, and *r*_*i*_ gives the rate of resource consumption of a mitochondrion of type *i* (*w* or *m*). The second term assigns a cost to the use of resource. The terms in this cost function are expressed as rates: *S* and *D* correspond to net energy production (supply) and demand per unit time. Supply and demand terms are left deliberately generalisable to encompass the differences in metabolic poise between cell types. The demand can be considered to represent energy requirements of all cellular processes besides mitochondria (whose maintenance costs are incorporated in their net supply *S*(*w*, *m*)), which we assume to be constant. We are therefore modelling post-mitotic cells in stable environments, as demands are expected to change throughout the cell cycle. This cost function can be evaluated for any state (*w*, *m*) and assigns the lowest cost to a state that satisfies demand in the most efficient way.

The net energy production of a state (*w*, *m*), *S*(*w*, *m*), is modelled as

$$\begin{array}{c} S\left(w,m\right)=w(s\left(r_{w}\right)-\rho_{1})+m(\epsilon_{2}s\left(\epsilon_{1}r_{w}\right)-\rho_{1})-\left(w+m\right)(\rho_{2}\lambda +\rho_{3}\mu ) \end{array}$$ (3)

where *ρ*_1,2,3_ are mitochondrial maintenance, building, and degradation costs, *s*(*r*_*w*_) denotes the power production (in ATP/s) of a single wildtype mitochondrion given a resource consumption rate *r*_*w*_ (Methods, section 4 in S1 File), and λ and *μ* (which can be functions of *w* and *m*) denote the birth and death rates in units per second. Mutant mtDNA molecules are distinguished by the parameters *ϵ*_1_, *ϵ*_2_ ∈ [0, 1] describing the mutant resource uptake rate (*ϵ*_1_) and efficiency (*ϵ*_2_) relative to that of the wildtypes (*r*_*m*_ = *ϵ*_1_*r*_*w*_). A low *ϵ*_1_ could represent reduced flow through the electron transport chain due to e.g. damaged respiratory complexes, whereas a low *ϵ*_2_ could denote increased proton leakage. Many mutants are known to have dysfunctional respiratory chain complexes [47], likely causing reduced electron flow through the respiratory chain and therefore reduced consumption rates of respiratory substrates such as NADH and oxygen. We therefore use *ϵ*_1_ < 1 and *ϵ*_2_ = 1 as our default choice (further described in section 4.7 in S1 File), though settings with *ϵ*_2_ < 1 are also investigated (section 5 in S1 File).

The more detailed structure of our cost function, which has been relatively general so far, comes from specifying the relation *s*(*r*_*w*_). In other words, how does the energy output of a mitochondrion depend on its resource (e.g. oxygen) consumption rate? We consider two possible forms for this function (section 3 in S1 File): a linear output relationship, suggested by some literature [48–50], and a saturating relationship, which accounts for finite resource consumption and spare mitochondrial capacity [51, 52] (section 5 in S1 File). We refer to these alternatives as the ‘linear output model’ and the ‘saturating output model’. Both models are described in more detail in our Methods section (Eq 7).

A trade-off between yield (efficiency) and rate of ATP production is present in yeast [53–55] whose rate of ATP production due to respiration can become saturated at high resource levels or limited oxygen supply [53, 56]. Higher energy production rates can then still be obtained by using fermentation at the expense of a lower yield [53]. A similar trade-off may exist in mitochondria, whose power production efficiency is higher when oxidizing NADH compared to oxidizing succinate [50]. The former may be the preferable substrate due to its higher yield, but if its levels become limiting an increase in the relative use of succinate would lower overall efficiency. When oxygen is limiting, increased glycolysis in an attempt to increase ATP production also leads to lower overall efficiency. These mechanisms could be the cause of a reduced power production efficiency at high energy demand, as proposed in our saturating output model. We will contrast our findings of the saturating output model with those generated by the linear output model.

We fitted the parameters of the linear output model using data provided in Ref. [48] (section 3 in S1 File), and set the parameters of the saturating model such that the two models behave similarly at low *r*_*i*_. Further details on the choice of parameter values, and their biochemical interpretations, are given in section 4 in S1 File; default values are provided in S2 Table. For a given demand *D* we find the *r*_*w*_ which gives a demand-matching supply (details of the scenarios when supply cannot meet demand are given in section Methods). Given *r*_*w*_ we calculate *r*_*m*_ (using *r*_*m*_ = *ϵ*_1_*r*_*w*_) and so calculate the cost *C*(*w*, *m*).

In taking both the linear and saturating output models into consideration we have endeavoured to build the most general picture of a mitochondrial cost function that retains bottom-up interpretability. Where possible, we estimate associated parameter values based on experimental data. However, other cost function choices are certainly possible and can be analysed using the platform we present below: our objective here is to complement the generic result regarding cost functions in paragraph “Nonlinear cost functions predict changes in tissue maintenance.” with a specific reasonable choice of cost.

#### Intermediate heteroplasmies may be inefficient and resource availability can dictate the cost of mtDNA states

**Fig 2:**
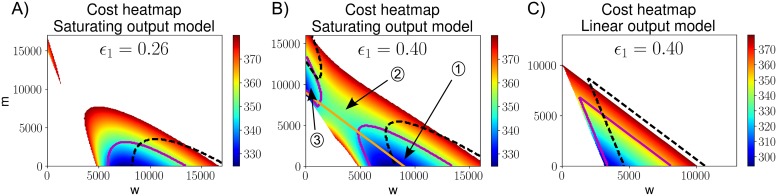
Intermediate heteroplasmies can be less efficient than either wildtype or mutant homoplasmy. A visualization of the cost function in (*w*, *m*) space is shown for both saturating and linear output models, for various mutant pathologies (described by *ϵ*_1_). For visualization purposes, states in which cellular demand cannot be satisified are shown in white. Cells in these states may still survive by e.g. increasing glycolysis (effectively reducing mitochondrial demand). This figure assumes high copy numbers, results are qualitatively similar for low copy numbers. The actual cost values (given by the colour map) are of lesser importance for our findings, we rather focus on the qualitative shape of the cost function. **A**: The magenta (solid) and black (dashed) lines show the contour of the demand-satisfying region when demand is increased by 10%, or demand is increased by 50% and cellular resource availability is increased by 35%, respectively. **B**: The orange line corresponds to constant total copy number; moving up along this line increases heteroplasmy. Cells in region 1 or region 3 are more efficient, and show a lower cost, than cells in region 2. **C**: The linear mitochondrial output model does not show a decreased efficiency at intermediate heteroplasmy values.

Fig 2 shows heatmaps of the cost function in (*w*, *m*)-space for different mutant pathologies (modelled as different values of *ϵ*_1_). Our cost function generates a heteroplasmy threshold, its value depending on both *w*_*opt*_ and *δ*, above which demand cannot be satisfied using oxidative phosphorylation (Fig 2), though increased glycolysis may still maintain cell viability. The threshold effect is an established phenomenon in mitochondrial physiology [7–14].

The state with lowest cost according to the linear output model is one with a minimum number of mitochondria required to satisfy demand (Fig 2C), where these mitochondria respire as fast as possible. This would mean that this state of lowest cost has no spare capacity. Assuming a cell controls its mitochondrial population towards the state with lowest cost, the linear model predicts cells to lack spare capacity, contradicting experimental observations [51, 57, 58]. A saturating output model solves this problem (S2E Fig and Fig 2A and 2B) and generates a trade-off between using each mitochondrion efficiently (minimising its resource consumption by increasing population) and minimising the cost of maintaining the total number of mitochondria (achieved by reducing the population). At low resource consumption, representing the linear regime of the saturating output model, the two models are similar.

We observe other qualitative differences in cost function structure between the saturating and linear output models. In the former, it is possible for intermediate heteroplasmy states to be more expensive than states homoplasmic for either species (Fig 2A and 2B). Hence, in the saturating output model, it is possible for intermediate heteroplasmies to be the least efficient and the most expensive (Fig 2 and S4 Fig, Table 1(III)). This result arises from a tradeoff, when mutant load is increased, between a decrease in global efficiency and a reduction in resource consumption by the new mutants (section 5 in S1 File). The linear output model, on the other hand, always shows higher costs at higher heteroplasmy (for fixed total copy number) (Fig 2C).

For our cost function, the existence of a high-cost intermediate heteroplasmy value is a relatively general feature of the saturating output model. We calculated the value of heteroplasmy with maximum cost (at constant total copy number), denoted by *h*_*max*_, as a function of several model parameters. *h*_*max*_ = 1 (the homoplasmic mutant state) at values *ϵ*_1_ ≲ 0.3 due to very low mutant functionality. However, at higher values of *ϵ*_1_ (0.5 ≲ *ϵ*_1_ < 1) we find *h*_*max*_ ∼ (0.5–0.8) over a large range of several of our cost function parameters (section 5.2 in S1 File). Though the size of the effect may be small, its existence alone is an interesting feature of our saturating output model.

It was previously found that it is possible for two mtDNA variants in mice to function normally at homoplasmy, but show deficiencies in heteroplasmic states [59]. While we do not claim that our model is the reason behind these observations it does suggest that differing resource consumption rates associated with distinct mtDNA species may play an important role.

### Combining cost and control: Comparison and optimisation of both cellular control and treatment strategies

#### Timescales and energy sensing in optimal control of mtDNA populations

Here we compare the mean cost over time for four plausible cellular control strategies. The first two consist of the linear feedback model λ(*w*, *m*) = *μ* + *c*_1_(*w*_*opt*_ − (*w* + *δm*)) with (I) *δ* = 0 (only wildtypes are sensed) and (II) *δ* = 1 (total mtDNA copy number is controlled). We further identify optimal parameterisations (i.e. ones that minimise steady-state cost) of two control strategies, namely (III) a linear feedback control and (IV) the ‘relaxed replication model’ (Eq (5)) [40, 41].

First, we fix the parameter values that are not being optimised. Our goal is to compare the costs of the dynamics resulting from each of the four controls, in the presence of mutants. In other words, we want to investigate whether some of these controls are better at protecting the cell (in the sense of maintaining a low energy cost) against mutant loads than others. To make this comparison fair, we demand that all controls yield the same dynamics in the absence of mutants: we set the wildtype mean and variance in this case to be identical under all controls. The mean is chosen to be *w*_*opt*_ (assuming that, without mutants, each control steers the population of wildtypes to its optimal value) and the variance is set by fixing the parameter *α*_*R*_ in the relaxed replication model to *α*_*R*_ = 10 (Eq (5)), as its value was originally estimated to lie in the range (5–17) [40]. This fixes the value for *c*_1_ in models (I)-(III) (S3 Table).

We now wish to optimise the parameters *δ* and *η* in models (III) and (IV). These parameters have direct impact on how mutants are sensed by the cell, and are important in determining how the dynamics change when mutant load increases. We thus want to investigate i) whether there exists an optimal amount of mutant sensing, and ii) how the cost of the dynamics resulting from these optimal parameters *δ*_*opt*_ and *η*_*opt*_ compares to that of our other two control strategies with *δ* fixed at either 0 or 1. To do this, we require both an optimization time-scale *T* and a set of initial conditions. We use *T* = ∞, corresponding to the steady state limit, and initial heteroplasmy values in the range *h*_0_ ∈ [0, 0.2]; we later consider finite values of *T*.

**Fig 3:**
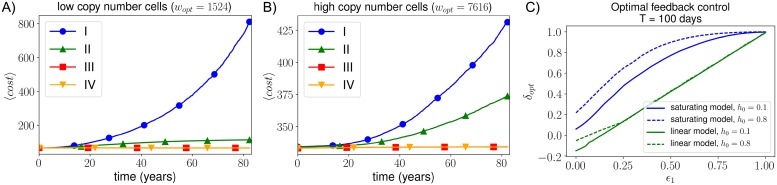
A control that senses no mutations shows an exponentially increasing cost, which is most noticeable in low copy number cells. **A + B**: Here we show the mean cost (3 × 10^4^ repeats) for the following four controls: linear feedback controls λ(*w*, *m*) = *μ* + *c*_1_(*w*_*opt*_ − (*w* + *δm*)) with I) *δ* = 0, II) *δ* = 1, and III) *δ* = *δ*_*opt*_, and IV) the optimised ‘relaxed replication control’ [40, 41] (Eq (5)). Controls were initialised in steady state at *h*_0_ = 0.15. Both figures used the saturating output model; figures (A) and (B) correspond to low and high copy number cells, respectively. We used *ϵ*_1_ = 0.3; other control parameters used are specified in section 5.3 in S1 File. **C: A control based on sensing mitochondrial energy output is generally a good strategy**. This plot shows the optimal value of *δ* in our linear control as a function of *ϵ*_1_, for the linear and saturating model and for both low (*h*_0_ = 0.1, solid line) and high (*h*_0_ = 0.8, dashed line) initial heteroplasmies. Here we used *T* = 100 and high copy number values for both models. Similar plots for *T* = 10^4^ are shown in S7 Fig, section 5.3 in S1 File. In the linear model *δ*_*opt*_ becomes negative for low *ϵ*_1_ values; as mutant copy number increases, a negative *δ* leads to an increase in wildtype to compensate for the deficient mutants.

Through stochastic simulations, we find that i) a control lacking any mutant contribution shows an exponential increase in cost over time, and ii) effects of particular control strategies are more pronounced in low copy number cells (Table 1(III)) (Fig 3). The relaxed replication rate control and our linear feedback function behave very similarly when *δ* and *η* take their optimal values. Cost variances, as well as mutant and wildtype dynamics, are shown in S6 Fig. Model II shows an increase in mean cost over time while mean mutant and wildtype copy numbers remain constant (Fig 3 and S6 Fig)—this is due to increases in copy number variances as argued previously.

We now investigate how the optimal value of mutant sensing for the linear control (*δ*_*opt*_) depends on timescale *T*, initial heteroplasmy *h*_0_ and the ‘mutant pathology level’ described by *ϵ*_1_. Here, we use the term ‘mutation pathology level’ to refer to a lower energy production rate of mutants due to a lower resource consumption rate, while ‘mutant sensing‘, as explained earlier, is used as a more general term. Intuitively, values of *ϵ*_1_ ≃ 1 have *δ*_*opt*_ ≈ 1: when wildtypes and mutants are equivalent, having a steady state with *w* + *m* = *w*_*opt*_ is desirable.

Values for *δ*_*opt*_ were found for the linear and saturating models, with low and high initial heteroplasmy values, for *T* = 100 days (Fig 3C). Having *δ* ≈ 1 means wildtypes and mutants are fed back similarly, whereas when *δ* ≪ 1 mutants are fed back less. For very deficient mutants (low *ϵ*_1_), a low *δ*_*opt*_ ensures that wildtype copy number remains close to its optimal value to compensate for the mutants. Generally, as *ϵ*_1_ decreases, *δ*_*opt*_ decreases (Fig 3C). Similar results are found for longer timescales *T* (section 5.3 in S1 File).

If mitochondrial energy outputs are sensed, the quantity ‘*w* + *δm*’ represents the mitochondrial energy production rate (power production). In this case, a mutant with low *ϵ*_1_ produces less energy and is thus sensed less (low *δ*). The relation between *ϵ*_1_ and *δ* now obeys the optimal trend shown in Fig 3C. Therefore, control strategies based on the energy status of the cell can often outperform controls based on mtDNA copy number (which always have *δ* = 1) or sensing mtDNA mass (which would work well for deficient deletion mutants, but would be suboptimal for deficient point mutations) (Table 1(IV)). Control based on copy number is preferred when a mutant is nearly as functional as a wildtype, in which case energy output and copy number are very much related. We have not used the expression for energy output in our cost function as a control strategy itself because claims based on the linear function *w* + *δm* are more general than one based on the details of our cost function.

#### Locally optimal control strategies map the control space of mtDNA populations

**Fig 4:**
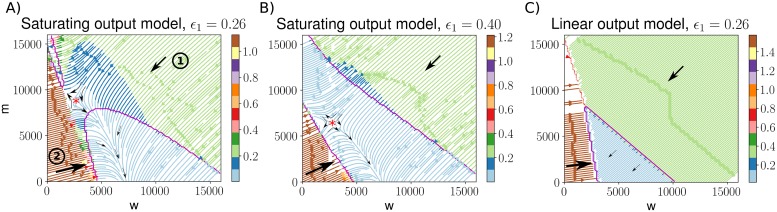
Locally optimal controls show nonlinear behaviours close to demand-satisfying regions, but linear optimal dynamics far away from these regions. Streamplots in (*w*, *m*)-space show the dynamics resulting from a locally optimal control, for various parameters of *ϵ*_1_. At each point, arrows show the direction corresponding to the largest decrease in cost. Regions are coloured according to the magnitude of the decrease in cost when moving in the optimal direction. Black arrows illustrate general trends in these regions. **A**: Region (1) shows that at high copy numbers, both mutant and wildtype mtDNAs should be decreased in an evenhanded manner; region (2) shows the possibility that the optimal control involves an increase in mutant copy number. **B**: A higher value for the parameter *ϵ*_1_ is used, meaning mutants are less pathological. **C**: the locally optimal control for the linear output model more closely resembles a linear control. In both (A) and (B) we see a divergence point (red asterisk) illustrating the fact that both high mutant and high wildtype states constitute local attractors of low cost (as in Fig 2).

Our cost function allows us to identify locally optimal controls: controls that, for each state (*w*, *m*), move the system in the direction of the largest decrease in cost. The resulting dynamics are shown in Fig 4. When heteroplasmy is high, the main priority is not always to decrease mutant copy number, but to increase wildtype copy number even if this means an increase in mutant load (region 2 in Fig 4A). Only after wildtype copy number has sufficiently increased should the focus be on decreasing *m*. At high copy numbers, the optimal dynamics are to decrease all mtDNA in an evenhanded manner (region 1) rather than decreasing *m* at a faster rate than *w*. For the saturating model, there is a divergence point in the space of local optimal strategies, reflecting the two local cost minima (high wildtype and high mutant) observed earlier (Fig 2). Hence, there are several regions of state space where even for pathological mutants, reduction of mutant mtDNA alone is not always the optimal control strategy (Table 1(V)). Finally, the less pathological the mutants become (e.g. Fig 4B), the more the locally optimal control starts to resemble a linear control. In the linear output model, the optimal control always shows linear behaviour (Fig 4C).

#### A parameterised model of artificial mtDNA control for disease treatment

In the previous section we identified locally optimal control strategies. Of course, these strategies may not be achievable by the cell (e.g. the cell may not be able to decouple biogenesis of wildtype and mutant mtDNA). However, such controls may still be possible through human intervention. This is why, in this section, we model recently developed genetic treatments to artificially control mtDNA populations. We then combine this treatment model with our linear feedback control λ(*w*, *m*) and our cost function.

Mitochondrially targeted zinc finger nucleases (mtZFNs) [35, 36, 60–62] are able to produce shifts in heteroplasmy by specifically cutting mutant mtDNA. To develop quantitative theory to understand and tune the effects of these interventions, we model nuclease transfection as inducing selective increases in mtDNA degradation, on the background of the linear cellular feedback control introduced earlier. Our transfection model contains three parameters describing strength (*I*_0_), duration (*b*), and selectivity (*ξ*) of nuclease treatment (Methods). We assume that for every mutant that is cleaved by the endonucleases, *ξ* wildtypes are cleaved [36]. For example, when *ξ* = 1 there is no distinction between mutants and wildtypes, and when *ξ* = 0 there is no off-target cleavage.

We fit these treatment parameters, as well as our feedback control parameters *c*_1_ and *δ*, to recently obtained experimental data [36]. These data involve heteroplasmy and mtDNA copy number measurements during iterative treatments with mtZFNs of 80% heteroplasmic human osteosarcoma 143B cybrid cells. Four sequential cycles of transfection and recovery were performed, where each recovery period lasted 28 days [36]. We use the data provided in Ref. [36] as well as additional data from this reference which was not explicitly provided in the paper. For the current study, we have collected new data consisting of: i) measurements of total mtDNA copy number in pre-treatment cells (which are used as initial conditions in our inference model), and ii) measurements of mtZFN expression profiles (S9 Fig).

#### A Bayesian description of nuclease treatment

**Fig 5:**
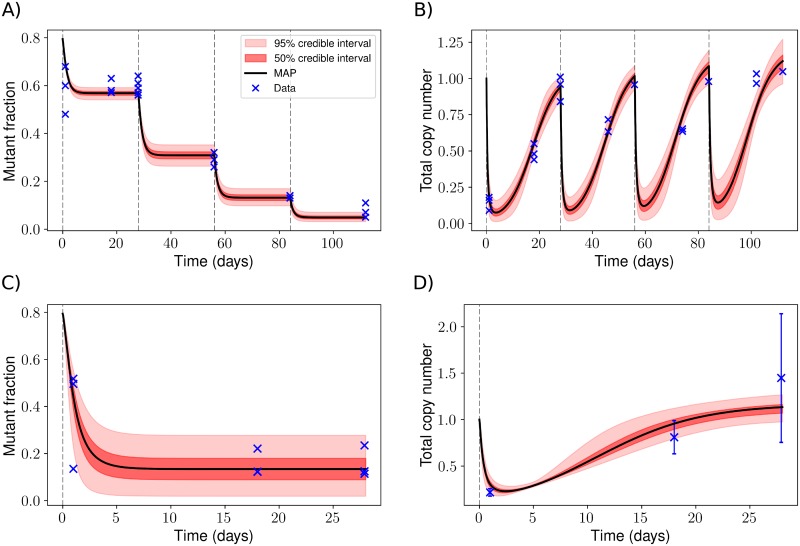
Bayesian credible intervals show the ability of a simple nuclease treatment model to capture experimental observations. We used Metropolis sampling to fit our model parameters to recently obtained experimental data [36]. Solid black lines correspond to the maximum a posteriori (MAP) prediction and vertical dashed lines indicate transfection events (once every 28 days). **A**: Drawing from our posterior distributions (5 × 10^4^ samples), we show the mean and 50% and 95% credible intervals of our heteroplasmy dynamics predictions during four rounds of treatment and recovery. Deterministic simulations were used. Crosses indicate data points from Ref. citeGammage16Near. **B**: Similar to figure (A), but showing relative total mtDNA copy numbers. **C + D**: Heteroplasmy and copy number dynamics were measured during a single round of treatment and recovery in a setting in which the mtZFN concentration was reduced by incorporating hammerhead ribozymes in the mtZFN backbone [36]. The credible intervals shown were obtained by sampling from the posterior distributions of the parameters *I*_0_, *b*, *c*_1_ and *δ* obtained using the data in figures (A) and (B), and using *ξ* ∼ 0.15 which represents the maximum likelihood estimate of *ξ* using this low mtZFN concentration data (Methods). Error bars in figure (D) show standard deviations of experimental measurements [36].

We use Metropolis sampling to obtain posterior distributions of the parameters *I*_0_, *b*, *ξ*, *c*_1_ and *δ* (Methods). Bayesian credible intervals for heteroplasmy and total copy number values during four consecutive rounds of treatment are shown in Fig 5A and 5B, illustrating the ability of this simple model to capture the dynamics resulting from nuclease activity. A periodicity of 28 days was imposed, representing the experimental protocol (Methods).

Our inference suggests the selectivity parameter *ξ* to lie in the range 0.6–0.8, indicating high levels of off-target cleavage (S10 Fig). This is not surprising given the large drop in total copy number (as low as ∼5% of initial values) combined with a modest shift in heteroplasmy (from 0.8 to ∼ 0.6) upon the first treatment. Supporting the high off-target cleavage, mtZFNs not targeted to any mtDNA sequence reduced copy numbers to 25% of their original values [36].

We now investigate whether our model can account for additional data (obtained in Ref. [36]) consisting of heteroplasmy and copy number measurements in a setting in which the concentration of mtZFNs is reduced by incorporating hammerhead ribozymes in the mtZFN backbone (for details, see Ref. [36]). A single round of treatment and recovery in this setting led to a large shift in heteroplasmy, from *h* ≈ 0.8 to *h* ≈ 0.2, and a drop in copy number similar to the previous setting in which mtZFN concentrations were higher (after 24 hours, mtDNA copy number dropped to ∼20% of its original value) [36]. These observations indicate that lower mtZFN concentrations lead to the treatment being more selective and, surprisingly, of similar strength. Because the additional data involves a different experimental setup inducing a large increase in selectivity, we adjust the parameter *ξ* to fit this additional data by finding its maximum likelihood estimate (Methods) but use posterior samples for all other parameters (obtained from inference based only on the data shown in Fig 5A and 5B). We find, consonant with an improved selectivity of this modified protocol, that *ξ* ≈ 0.15 in this low mtZFN concentration setting (Methods) and that our model can reproduce the heteroplasmy and copy number dynamics using our previously fitted parameters *I*_0_, *b*, *c*_1_ and *δ* (Fig 5C and 5D).

Finally, we measured transient expression profiles of mtZFNs using the same transfection protocol as in Ref. [36] (S9 Fig). Posterior samples of the parameters *I*_0_ and *b* predict that mtZFN levels have dropped to very low levels 5 days post-transfection in the setting without hammerhead ribozymes, consistent with our obtained experimental data (S11 Fig). We thus show that our model is capable of capturing the dynamics of several data sets. Our mtZFN treatment model predicts that total copy number reaches a minimum at around 3 days (Fig 5B and 5D).

#### Knowledge of the heteroplasmy distribution of a tissue is important in determinining how effciently the tissue can be treated

**Fig 6:**
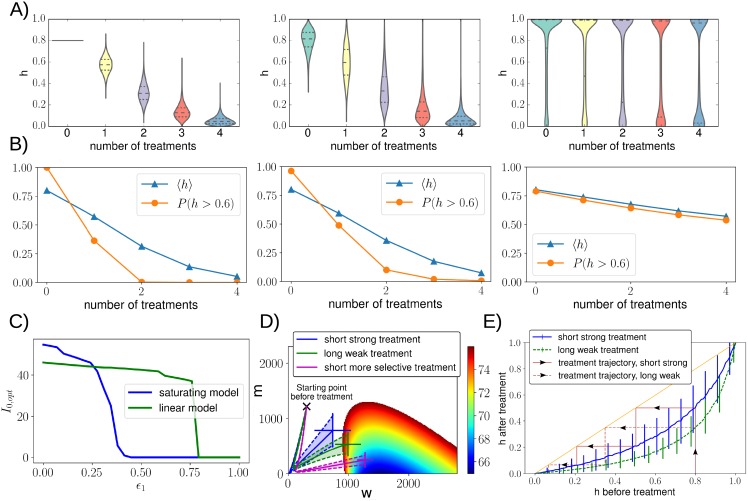
Knowledge of the heteroplasmy distribution is important in predicting how efficiently a tissue can be treated. **A + B**: The effect of four simulated consecutive treatments on three different initial heteroplasmy distributions is shown; all initial distributions have identical means (〈*h*〉 = 0.8) but different variances (increasing from left to right). The higher the variance of the initial population, the harder to shift mean heteroplasmy values; mean values after each treatment as well as *P*(*h* > 0.6) are shown in figure (B). In these simulations we assumed that every cell gets transfected. **Gentle but sustained treatments induce larger heteroplasmy shifts than hard and brief treatments. C**: Both the linear and saturating model show a sharp drop in the optimal treatment strength *I*_0,*opt*_ as the mutants become more functional (i.e. as *ϵ*_1_ increases). **D**: Means and variances of mutant and wildtype copy numbers were simulated during a round of treatment and recovery, using: i) parameters fitted to the data shown in Fig 5A and 5B (blue), ii) a longer treatment duration (smaller *b*, green) and iii) a higher selectivity (smaller *ξ*, magenta). MtZFN levels first drop below 5% of their maximum values after ∼4.5 and ∼35 days for the short (blue, magenta) and long (green) treatments, respectively. The longer weaker treatment induces higher heteroplasmy shifts than the shorter stronger treatment. *ϵ*_1_ = 0.2 was used, the corresponding cost heatmap is shown. Error bars show standard deviations (based on 10^4^ stochastic simulations), further detailed are given in section 6.6 in S1 File. **E**: This figure also illustrates that gentle sustained treatments lead to larger heteroplasmy shifts. Examples of treatment trajectories are shown; after a single treatment, an initial heteroplasmy of 0.8 is mapped to 0.53 (short strong treatment) or 0.39 (long weak treatment). Parameters chosen in figures (A)–(E) are based on the inference performed earlier, their exact values are provided in section 6.6 in S1 File.

To explore the effect of the heteroplasmy distribution on treatment efficacy, we consider three initial *h* distributions with different variances but identical homogenate means. We treat these populations multiple times using the parameter fits obtained in the previous section. The resulting shifts in heteroplasmy distribution, including mean and threshold-crossing probability, are shown in Fig 6A and 6B. High heteroplasmy variances require many cells close to the two extremes *h* = 0 and *h* = 1, which are challenging to shift. A striking reduction in treatment efficacy is predicted as heteroplasmy variance increases while fixing its mean (Fig 6A and 6B). Threshold crossing probability (for example, *P*(*h* > 0.6)) also becomes harder to shift at higher variance. We conclude that tissues with a high mean heteroplasmy level will generally be harder to treat if the heteroplasmy variance is high, especially if this high mean level is caused by a small percentage of cells.

We can use our parameterised theory to find optimal treatment strengths *I*_0,*opt*_ for a given system. Fig 6C shows *I*_0,*opt*_ as a function of *ϵ*_1_. Intuitively, the strongest treatment should be given to the least functional mutants, and when mutants are almost as functional as wildtypes it is preferable not to treat at all. The optimal treatment strength drops rather sharply as *ϵ*_1_ increases, and does so sooner for the saturating model. This last observation may be because at some point reducing heteroplasmy becomes more expensive as can be seen in Fig 2B. Optimal treatment strengths for longer treatments (higher *b*) show similar qualitative behaviour.

Fig 6D shows trajectories in (*w*, *m*) space throughout a single treatment and recovery phase. The three trajectories shown correspond to: i) a short and strong treatment, ii) a long and weak treatment, and iii) a short but more selective treatment. The value for *I*_0_ was chosen such that, for the specific treatment duration *b* used and given a fixed total simulation time, the shift in heteroplasmy was largest. For the short treatment a relatively large proportion of time is spent fluctuating around steady state values (dynamics which do not change mean *h*) due to a relatively quick recovery, whereas for longer treatments more time is spent in the treatment phase itself (dynamics which lower mean *h*). We thus find that in a given time frame, treating longer but weaker results in a lower final heteroplasmy value than treating short and strong (Table 1(VIII)). A weaker treatment also reduces the chance of a cell losing all its mtDNA molecules. Intuitively, a more selective treatment leads to larger heteroplasmy shifts.

Nuclease treatment and a subsequent recovery phase will have the net effect of mapping an initial heteroplasmy value *h*_*i*_ to a mean final heteroplasmy value, *h*_*f*_. We simulated this mapping in the presence of cellular feedback control (Fig 6E), finding that heteroplasmy shifts are largest for intermediate heteroplasmies. The difference in treatment results for long compared to short treatments is also illustrated. Interestingly, for high *h* values, it is possible to end up with a **higher** heteroplasmy value after treatment, especially if *ξ* ≃ 1 (S8 Fig).

## Discussion

In this work, we have built a quantitative theory bridging stochastic optimal control, costs of mtDNA populations, and gene therapies. Our results contribute to a growing body of evidence [63–66] that the variance of mtDNA populations has important physiological and therapeutic implications independently of mean heteroplasmy, and underline that stochastic theory is required to understand this biologically and medically important quantity.

Key findings of our model (Table 1) include (I) the identification of tradeoffs in the control of one or the other mtDNA species; (II) the observation that increasing mtDNA variance can lead to increased energetic costs over time and ageing even when means and demands are preserved; (III) intermediate heteroplasmy states can be more expensive than states homoplasmic in either mutant or wildtype; (IV) mutant sensing can be required to avoid an exponentially increasing cost; (V) sensing of cellular energetic status can be more effective than other targets like mitochondrial mass; (VI) reduction of mutant mtDNA alone is not always the optimal control strategy; (VII) high heteroplasmy variance challenges gene therapy treatments; and (VIII) weak, long gene therapy trajectories are more effective than short, intense ones.

Our findings hold qualitatively under the range of conditions we discuss above. The aim of our manuscript is not to make detailed quantitative predictions and conclusions based on complex models, nor do we intend to imply that our models are the only possible models one could construct. Rather, we aim to provide general biologically plausible models to gain qualitative insights and to comment on large-scale behaviours. To this end, our cost function, used to illustrate some of our results, is phenomenological and contains several parameters. Most of these are biologically interpretable, meaning their values can be obtained or estimated from the literature. The main elements in our cost function are quite general: terms involving supply, demand, and resource.

To test the qualitative shape of our cost function, one could sort cells based on mitochondrial copy number and heteroplasmy to obtain samples at different points in (*w*, *m*) space. Measurements of e.g. cell proliferation, ROS or apoptosis rates allow for the evaluation of an effective cost at each of these points. By measuring the relative consumption rates of NADH and succinate, as well as the amount of ATP produced per glucose consumed, in identical cells exposed to different energy demands, the saturating output model may be probed.

If the parameter *δ* is low, i.e. mutants are sensed less, mutant copy numbers at high heteroplasmies will be higher than wildtype copy numbers at low heteroplasmies. Experimentally, it has been observed that heteroplasmic cells can have total mtDNA copy number values that are 5-17-fold higher compared to cells homoplasmic in wildtype [67–70]. The cell has somehow allowed these mutants to expand, which may mean that they are less tightly controlled; controls based on total energy output or mtDNA mass (which can result in *δ* < 1) may lead to such behaviours. A control on mtDNA mass could explain why deletion mutants are often seen to expand [71, 72] and would also predict normal copy number levels in cells harbouring mtDNA point mutations. Recently, it was found that samples with mtDNA indels had very high mtDNA copy number levels, but single nucleotide variants did not [73].

We showed that heteroplasmy distributions in cell populations can provide important information about the possibility of successfully treating these cells with endonucleases. A tissue may be harder to treat if its high mean heteroplasmy level is caused by a small percentage of dysfunctional cells. Experimental values of mean homogenate heteroplasmy in heart tissue of patients with the 3243A>G mutation are roughly around 0.8 (though ranges can be large [74–77]) and muscle tissue often shows mosaic structures, with deficient patches of cells adjacent to healthy cells. These examples show that it may be that, at least in some cases, high mean levels are indeed caused by a relatively low percentage of cells, meaning that there are still challenges ahead for efficiently treating these tissues.

One of the features of our cost function is that resource limitations play an important role in shaping the cost landscape. There are indications that cellular levels of NAD (a coenzyme involved in oxidative phosphorylation) are limiting, and that a sufficient supply of NAD to mitochondria becomes critical [78–81]. An increase of intracellular NAD can lead to an increase in oxygen consumption and ATP production [81] indicating that resource limitation may, at least in some cases, be a genuine constraint. Adding various kinds of resources can significantly change mitochondrial basal respiration rate [82–84].

Like any other model, our models have a defined range of applicability. A key baseline assumption was using identical replication and degradation rates for mutants and wildtypes. Various possibilities of distinct rates have been offered in the literature, including faster mutant replication rates [22, 68, 85–88], lower mutant degradation rates [89], and higher mutant degradation rates [90, 91]. Including such differences, and other features such as *de novo* mutations, degradation control, and cell divisions [38, 64, 92, 93], constitute natural extensions to our theory.

## Methods

### Wildtype and mutant mtDNA evolution equations

Wildtype and mutant mtDNA copy numbers are considered to have birth rate λ(*w*, *m*) = *μ* + *c*_1_(*w*_*opt*_ − (*w* + *δm*)) and death rate *μ*, leading to the following evolution equations:

$$\begin{array}{cc} \frac{dw}{dt} & =w(\lambda (w,m)-\mu )\\ \frac{dm}{dt} & =m(\lambda (w,m)-\mu ) \end{array}$$ (4)

The corresponding stochastic system, required to e.g. describe fixation, does not have an explicit solution due to nonlinearities. The deterministic steady state solution of Eq (4) is given by (*w*_*ss*_ + *δm*_*ss*_) = *w*_*opt*_ and represents a straight line in (*w*, *m*)-space (S1A Fig), whose slope depends on the value of *δ*. Stochastic dynamics will fluctuate around the steady state line, causing heteroplasmy to change over time until fixation of either species occurs. This means that, over long times, a cell will reach either *h* = 0 or *h* = 1 (in the absence of mutations). When mutations do occur, a cell will always reach a state with *h* = 1 (though many different mutant species may be present).

### Relaxed replication model

The relaxed replication model assumes a constant death rate *μ* and a birth rate of the form

$$\begin{array}{c} \lambda \left(w,m\right)=\frac{\mu }{w+m}(\alpha_{R}[w_{opt}-\left(w+\eta m\right)]+w+\eta m) \end{array}$$ (5)

with *α*_*R*_ > 1 and *η* constants [40, 41]. We have renamed the parameters of the original model for convenience. Note that both *α*_*R*_ and *η* influence the mutant contribution to λ(*w*, *m*) (rather than the single parameter *δ* in our linear model).

### Expected cost per unit time

Let the cost per unit time of state (*w*, *m*) be denoted by *C*, and the cost corresponding to the steady state (*w*_*ss*_, *m*_*ss*_) by $\bar{C}$. Even if steady state copy numbers are constant over time (i.e. the mean values of *w* and *m* are always equal to *w*_*ss*_ and *m*_*ss*_) the mean cost per unit time is generally not equal to $\bar{C}$. By performing a Taylor expansion, the mean cost per unit time can be written as follows:

$$\begin{array}{cc} E[C](t) & \approx \bar{C}+\frac{1}{2}(\text{var}\left(w\left(t\right)\right)\frac{\partial^{2}C}{\partial w^{2}}+\text{var}\left(m\left(t\right)\right)\frac{\partial^{2}C}{\partial m^{2}}\\ & +2\text{cov}\left(w\left(t\right),m\left(t\right)\right)\frac{\partial^{2}C}{\partial w\partial m}) \end{array}$$ (6)

where *E*[*C*](*t*) is the expected cost per unit time given that the trajectory starts in state (*w*_*ss*_, *m*_*ss*_), and all partial derivatives are evaluated at steady state. These findings imply the following: suppose all cells in a population of cells are initialised in a state with minimum cost (corresponding to some specific number of mutant and wildtype mtDNA molecules). At some later time, the mtDNA populations in the different cells will have drifted apart and even if mean copy numbers (averaged over all cells) of *w* and *m* are identical to their initial values, the increase in variance between cells means that the overall mean cost (averaged over all cells) is higher than it was initially.

### Cost function structure

We assume that the net energy supply per unit time in a state (*w*, *m*), called *S*(*w*, *m*), involves the following four terms: (i) the energy output per unit time (*s*_*i*_) produced by the mitochondria; (ii) a maintenance cost per unit time (*ρ*_1_) to maintain the mitochondria, as their presence imposes some energetic cost (e.g. mRNA and protein synthesis); (iii) a building cost (*ρ*_2_) for the biogenesis of new mitochondria; and (iv) a degradation cost (*ρ*_3_) to degrade mitochondria. We will assume that every mtDNA molecule is associated to a particular amount of mitochondrial volume which we refer to as a ‘mitochondrion’ (section 4 in S1 File).

At any time, mitochondria experience a certain energy demand and to meet this demand they need to have a certain resource consumption rate *r*_*i*_ (where *i* = *w*, *m* refers to wildtype or mutant). Here we use the term ‘resource’ as an amalgamation of the substrates used for the oxidation system. We need to specify the relationship between the power supply (*s*) and the rate of resources consumed (*r*_*i*_) by mitochondria. We use two different models *s*(*r*_*i*_) which are discussed further in section 3 in S1 File

$$\begin{array}{cc} s(r_{w}) & =\phi (r_{w}-\beta )\\ s(r_{w}) & =2\frac{s_{max}}{1+e^{-kr_{w}}}-1.1s_{max} \end{array}$$ (7)

where *ϕ*, *β*, *k* and *s*_*max*_ are constants respectively describing the mitochondrial efficiency, a basal proton leak-like term, the saturation rate of the efficiency, and the maximum power supply (section 4 in S1 File).

We assume that pathological mutants can have a deficient electron transport chain (which may support a smaller flux leading to a lower resource consumption rate for mutants and therefore a lower ATP production rate) and a lower energy production efficiency, leading to the following mutant energy output: *ϵ*_2_*s*(*ϵ*_1_*r*_*w*_). Here, *ϵ*_1_, *ϵ*_2_ ∈ [0, 1] describe the mutant resource uptake rate and the mutant energy production efficiency relative to that of a wildtype, respectively. In the main text we set *ϵ*_2_ = 1; other values of *ϵ*_2_ are discussed in section 4.7 in S1 File.

The mitochondrial maintenance cost is denoted by *ρ*_1_ and corresponds to the energetic cost required to maintain the mitochondrion that contains the mtDNA. This energetic costs involves factors like the synthesis and degradation of mitochondrial proteins and enzymes. We assume the maintenance cost is the same for wildtype and mutant mitochondria (though for some mutations this is quite possibly not the case). The net energy supply per unit time, *S*(*w*, *m*), then follows as Eq 3.

To determine the value of *r*_*w*_ for a given state (*w*, *m*), we first check whether the demand *D* (which we assume is a constant) can be satisfied by supply *S*(*w*, *m*). If it can, we set Eq (3) equal to *D* and solve for *r*_*w*_, i.e. we assume that if possible, the mitochondria will exactly satisfy demand. It may, however, not be possible to satisfy demand, which can be because of two reasons: i) there are not enough mitochondria present to produce enough energy, or ii) the resource supply rate, *R* (a constant), is not enough to meet demand. In the former case, we set *r*_*w*_ = *r*_*max*_ (a specified maximum resource consumption rate per mitochondrion): the mitochondria work as hard as possible to keep their energy output closest to demand. In the latter case, we assume that the total available resource supply is shared equally between the mitochondria: $r_{w}=\frac{R}{w+\epsilon_{1}m}$. Further details of the cost function are given in sections 3–5 in S1 File.

The parameters used in our cost function are summarised in S2 Table and motivated in section 4 in S1 File. Despite our model being simple, most parameters are biologically interpretable.

### Modelling control through mitochondrially targeted endonucleases

Experimentally, cells are transfected with two mtZFN monomers: one which binds selectively to mutant mtDNAs, and one that binds mutants and wildtypes with equal strength [62]. We simplify this picture by assuming an ‘effective’ mtZFN pool and use [*ZFN*] to denote its concentration. The increase in mtDNA degradation rate caused by the mtZFNs is then assumed to be proportional to [*ZFN*].

Nucleases are imported into the cell and then degrade over time, meaning that their concentration in the cell (and in the mitochondria) may be approximated by an immigration-death model:

$$\begin{array}{c} \frac{d[ZFN](t)}{dt}=I\left(t\right)-\mu_{z}\left[ZFN\right]\left(t\right) \end{array}$$ (8)

where *I*(*t*) and *μ*_*Z*_ are the immigration and death rates of the effective mtZFN pool, respectively. In recent experiments [36], nucleases are expressed for short times meaning that the immigration rate will increase sharply at the start of the treatment after which it decreases over time: we chose to model *I*(*t*) as an exponentially decaying function, *I*(*t*) = *I*_0_*e*^−*bt*^, where *I*_0_ denotes the initial rate directly after the treatment is initiated and *b* is a constant describing the duration of the treatment. The mtZFN concentration now becomes

$$\begin{array}{c} \left[ZFN\right]\left(t\right)=\frac{I_{0}}{\mu_{z}-b}(e^{-bt}-e^{-\mu_{z}t}) \end{array}$$ (9)

which is shown for various parameter values in S8A Fig. The data we use to fit our models concerns heteroplasmy and total copy number measurements over four rounds of treatment, each treatment consisting of mtZFN transfection followed by a 28-day recovery period. During this recovery period, total copy numbers recover their initial values due to cellular feedback control. The increase in mtDNA death rate due to the presence of the mtZFNs, *μ*_*ZFN*_, is given by

$$\begin{array}{c} \mu_{ZFN}\left(28\cdot i<t<28\cdot \left(i+1\right)\right)=\mu +\sum_{j=0}^{i}\left[ZFN\right]\left(t-28\cdot j\right) \end{array}$$ (10)

where *i* = 0, 1, 2, 3 indicates the treatment round. This equation is simply stating that new mtZFNs are added every 28 days. Death rates for *m* and *w* are now assumed to be

$$\begin{array}{cc} \mu {\left(t\right)}_{w} & =\mu +\xi \cdot \mu_{ZFN}\left(t\right)\\ \mu {\left(t\right)}_{m} & =\mu +\mu_{ZFN}\left(t\right) \end{array}$$ (11)

where *μ* denotes the baseline degradation rate and *ξ* represents treatment selectivity (e.g. when *ξ* = 0 there is no off-target cleavage).

### Model fits using Metropolis sampling

To fit our nuclease model to recently obtained experimental data [36], we use Eq (4) with *μ* replaced by *μ*(*t*)_*w*_ or *μ*(*t*)_*m*_ and λ(*w*, *m*) given by Eq (1):

$$\begin{array}{cc} \frac{dw}{dt} & =w[c_{1}\left(w_{opt}-\left(w+\delta m\right)\right)-\xi \cdot \mu_{ZFN}\left(t\right)]\\ \frac{dm}{dt} & =m[c_{1}\left(w_{opt}-\left(w+\delta m\right)\right)-\mu_{ZFN}\left(t\right)] \end{array}$$ (12)

Total mtDNA copy numbers in pre-treatment 80% heteroplasmy cells were measured using quantitative PCR (section 6.4 in S1 File) and were found to be 889 ± 214 (S.E., *n* = 3). We therefore assume an initial total copy number of 900, meaning *w* and *m* were initialized at 0.2 ⋅ 900 = 180 and 0.8 ⋅ 900 = 720, respectively. These evolution equations incorporate cellular feedback control as well as the nuclease treatment which occurs in cycles of 28 days. The mtZFN degradation rate was assumed to be *μ*_*z*_ = ln(2) day^−1^, corresponding to a half-life of 1 day. This is in accord with the experimental observation that almost no mtZFN was present 4 days post-transfection (with a half-life of 1 day, only 6% of initial copy numbers remain after 4 days).

MCMC inference was performed using the Python package Pymc3, a package designed for Bayesian statistical modelling and probabilistic machine learning [94]. A Gaussian error model was assumed, i.e. the observed heteroplasmy $y_{i}^{\left(h\right)}$ and total copy number $y_{i}^{\left(T\right)}$ data are given by

$$\begin{array}{cc} y_{i}^{\left(h\right)} & ={\hat{y}}_{i}^{\left(h\right)}+N\left(0,\sigma_{h}^{2}\right)\\ y_{i}^{\left(T\right)} & ={\hat{y}}_{i}^{\left(T\right)}+N\left(0,\sigma_{T}^{2}\right) \end{array}$$ (13)

where ${\hat{y}}_{i}^{\left(h\right)}$ and ${\hat{y}}_{i}^{\left(T\right)}$ denote our predicted heteroplasmy and copy number values obtained by numerically solving Eq (12), and we allow for different noise variances for *h* and *T* (in general, different experimental errors are expected as different methods are used to measure *h* and *T*). A metropolis sampler is used for parameter estimation. Maximum a posteriori (MAP) values were found to be ${\left(I_{0},b,c_{1},\xi ,\delta ,\sigma_{h}^{2},\sigma_{T}^{2}\right)}_{MAP}\approx \left(122.82,46.68,1.90\times 10^{-4},0.72,1.26,0.061,0.10\right)$. Due to a degeneracy in our mtZFN dynamics model (section 6.5 in S1 File) the MAP values of *I*_0_ and *b* are not necessarily unique at large *b* (details in section 6.5 in S1 File).

We explore the ability of our model to account for additional data from Ref. [36] (Fig 5C and 5D) which was not included in our inference. Using the MAP values for parameters *I*_0_, *b*, *c*_1_, *δ*, $\sigma_{h}^{2}$ and $\sigma_{T}^{2}$ (based on the data shown in Fig 5A and 5B), the maximum likelihood estimate of *ξ* is obtained based on the additional data, using a Gaussian error model similar to Eq (13). This maximum likelihood value is *ξ* ≈ 0.15.

## Supporting information

S1 Fig A linear feedback control has straight steady state lines. **A)** The deterministic steady state lines of the feedback control given in Eq 4, using our linear version of λ(*w*, *m*), are shown in (*w*, *m*) space for various values of *δ* (grey lines show particular examples of ranges of *δ*). Constant heteroplasmy lines form straight lines through the origin. **B, C, D) Equal variances for different feedback control mechanisms**. Three different controls (see legend), all of the form λ(*w* + *δm*) with *δ* = 0.5, show nearly identical wildtype, mutant and heteroplasmy variances. Other parameters used are *N*_*ss*_ = 1000 (referring to the steady state copy number present in the absence of mutants), *μ* = 0.07 (corresponding to a half-life of 10 days), and initial copy numbers (*w*_0_, *m*_0_) = (920, 160) (corresponding to an initial heteroplasmy of ∼ 0.15).
(EPS)

S2 Fig Relationship between resource consumption and energy output. **A)** The energy production rate of a single wildtype mitochondrion as a function of its resource consumption rate is shown, as given by Eqs. (15) and (16) in S1 File. For the linear model (corresponding to the straight lines) the parameters *ϕ* and *β* are changed by 10%, for the saturating model we vary *s*_*max*_ and *k*. The magenta line indicates *r*_*max*_. **B)** As *w* increases, demand is shared between more mitochondria and each individual one can afford to consume resources at a lower rate (the same figure legend applies for figures C, D and E). **C)** The total resource consumption increases with *w* because the mitochondria need to consume a non-zero amount of resources to produce a net energy output and each mitochondrion comes with a maintenance cost. **D)** The total energy produced by wildtypes increases when mutants are present. **E)** When demand is satisfied, the cost increases with *w* in the linear model, resulting in minimal costs when copy numbers attain the minimum number required to satisfy demand (1). In contrast, for the saturating model the cost decreases at first because as individual resource consumption drops, the energy production efficiency increases. Minimum cost now occurs when mitochondria are working most efficiently (2). Parameters *ϵ*_1_ = 0.1 and *ϵ*_2_ = 1.0 were used.
(EPS)

S3 Fig Changing mutant efficiency (*ϵ*_2_) does not lead to expensive intermediate heteroplasmies. **A), B)** Similar to Fig 2 in the main text, these figures show the cost values in (*w*, *m*) space, but now as a function of *ϵ*_2_ (mutant efficiency) instead of *ϵ*_1_. This time we show the cost in the entire space. The white lines show the region in which demand is satisfied for our default parameter values. Because mutants consume the same amount of resource as wildtypes (*ϵ*_1_ = 1), resource becomes limiting at relatively low values of *m* compared to when *ϵ*_1_ < 1. Note that intermediate heteroplasmies are not less efficient here.
(EPS)

S4 Fig Intermediate h values require more resources to satisfy demand, but only if mutants consume less resources. **A)** The resource consumption rates and energy production rates of wildtypes and mutants are shown for two states: (*w*_1_, *m*_1_, *h*_1_) = (9000, 1000, 0.1) and (*w*_2_, *m*_2_, *h*_2_) = (7000, 3000, 0.3). In both cases, the total energy output is equal to the demand. When heteroplasmy is higher (*h* = 0.3), the individual resource consumption rates are higher in order to maintain a constant total energy output. Overall, the state with *h* = 0.1 uses the least resources (Eq. (20) in S1 File). *ϵ*_1_ = 0.35 was used. **B)** This figure is similar to figure (D) but now the two states (*w*_1_, *m*_1_, *h*_1_) = (3000, 7000, 0.7) and (*w*_2_, *m*_2_, *h*_2_) = (1000, 9000, 0.9) are compared. The state with *h* = 0.9 uses the least resources (Eq. (21) in S1 File).
(EPS)

S5 Fig The existence of intermediate heteroplasmy values is a robust feature of the saturating output model. We show the value of *h*_*max*_, the most expensive heteroplasmy value at constant copy number, as a function of total copy number and *ϵ*_1_ (describing mutant pathology). White regions correspond to *h*_*max*_ = 1. **A)** Using our default parameter values, an intermediate *h*_*max*_ exists for large enough mutant functionality *ϵ*_1_. **B)** The parameter *s*_*max*_ is increased by 50% with minimal effect on the output. We kept the parameter *k* fixed as it defines the amount of proton leak (the resource consumption rate at zero energy output) which agrees with the amount of proton leak in the linear output model whose parameters are based on experimental data. **C)** The parameter *ρ*_1_ is increased by an order of magnitude with minimal effect on the output. **D)** The parameter *ρ*_1_ is increased by an order of magnitude and *s*_*max*_ is decreased by 50%. Again, change in *h*_*max*_ are small.
(EPS)

S6 Fig Wildtype, mutant and cost dynamics for four different control strategies. Dynamics are shown for the four controls I, II, III, and IV defined in Table 3 in S1 File. Again, we see that the effects of the control are more noticeable in low copy number cells. Parameters are set as given in Table 3 in S1 File. Values for *w*_*opt*_ are those for the saturating output model at low and high copy number. The free parameters in control III and IV (*δ* and *η*) were optimised over initial conditions in the range *h* ∈ [0, 0.2]. For the optimization the default cost function parameters were used as well as *ϵ*_1_ = 0.3.
(EPS)

S7 Fig At long times and high heteroplasmies, energy sensing control becomes suboptimal. The optimal value of *δ* in a linear feedback control is shown as a function of *ϵ*_1_. Here we used *T* = 10^4^ days (optimization time) and low copy numbers for both the linear and saturating model. The solid and dashed lines correspond to trajectories starting at *h*_0_ = 0.1 and *h*_0_ = 0.8, respectively. The less resources the mutants consume (and the less output they therefore produce) the lower their optimal contribution to the control.
(EPS)

S8 Fig Zinc finger nuclease concentrations for short and long treatments. **A)** Here we show the concentration of mitochondrially targeted Zinc Fingers as modelled by Eq (9) in the main text. The parameter values for the short and strong treatment illustrated here (*I*_0_ = 36, *b* = 11) are similar to those found in fitting the model to the data. For the mtZFN degradation rate we used *μ*_*Z*_ = log(2) day^−1^ (corresponding to a mtZFN half-life of 1 day). **There exists a possibility of increasing heteroplasmy levels through treatment**. **B)** The probability of increasing heteroplasmy above its initial pre-treatment value *h*_0_, after one round of treatment and recovery, is shown as a function of *h*_0_ and *ξ*. Cells are initialised with a total copy number of 500. The cross indicates the parameters used in figure (D). The parameter values for *I*_0_, *b* and *c*_1_ are fixed at: (*I*_0_, *b*, *c*_1_) ≈ (39, 20, 3 × 10^−4^); these values provide good fits to experimental data when assuming a total initial copy number of 500. We used *δ* = 1. **C)** Similar to figure (B), but now cells are initialised with a total copy number of 5000; in these large copy number cells stochastic fluctuations in copy number have less effect and the probabilities of exceeding initial heteroplasmy values are smaller compared to figure (B). **D)** An example of a distribution of post-treatment heteroplasmy values is shown using parameters *h*_0_ and *ξ* as indicated by the cross in figure (B). The orange line indicates the value of *h*_0_ (the heteroplasmy that was present before the treatment started).
(EPS)

S9 Fig mtZFN expression profile during transient transfection of 143B cells. **A)** Here we show a schematic of the experiments involving i) transient transfection of high-heteroplasmy cells with plasmids expressing mtZFN monomers and fluorescent marker proteins, ii) FACS-based selection of cells expressing both mtZFN monomers (NARPd(+) and COMPa(-)), and iii) phenotypic evaluation of treated cells. Technical details are provided in Ref. [35]. **B)** Western blots showing the mtZFN expression profile indicate that the mtZFNs are almost undetectable at 96 hours post-transfection, and completely undetectable at 120 hours. Details of the protocol are provided in Ref. [35].
(EPS)

S10 Fig Posterior mtZFN treatment parameter distributions. Here we show our posterior distributions obtained after running our MCMC algorithm (left) as well as the corresponding sample values (right). Prior distributions are provided in the text. The posterior of log_10_ *b* is cut off due to a degeneracy in our model (Eq. (22) in S1 File), which does not affect our model predictions.
(EPS)

S11 Fig Predictions of mtZFN expression are broadly consonant with experimental data. Drawing from our posterior distributions for *I*_0_ and *b* obtained through Metropolis sampling, we show 50% and 95% credible intervals of our predicted mtZFN expression profile (solid black line denotes the maximum a posteriori (MAP) estimate). Data points are obtained through quantification of the western blots shown in S9B Fig and were subsequently rescaled to investigate whether our model can broadly account for the experimentally observed dynamics (our predicted mtZFN concentrations are proportional to the measurement data points with an arbitrary proportionality constant).
(EPS)

S1 Table Analytical expressions for the means and variances according to the linear noise approximation. Solutions are shown for wildtype, mutant, and heteroplasmy variances for various types of control. Dots indicate constant or exponentially decaying terms; full solutions are provided in Eqs. (11)–(13) in S1 File. Note that the initial rate of increase of heteroplasmy variance only depends on mtDNA copy number and turnover (see also [38]).
(EPS)

S2 Table Parameters used in our mitochondrial cost function with their descriptions. Parameter values are derived and motivated in Section 4 in S1 File. In this table, ‘high’ and ‘low’ refer to high and low copy numbers, respectively, and the abbreviations ‘sat.’ and ‘lin.’ are used to indicate our saturating and linear output models.
(EPS)

S3 Table Parameter values for the four different control mechanisms we employ. Two parameters of each control are set by the two constraints we impose. The parameter *α*_*R*_ was proposed to lie in the range 5-17 [40] and here we used *α*_*R*_ = 10. The values for *δ* and *η* are found by optimizing our cost function over the steady states corresponding to our initial conditions. We used 50 initial conditions equally spread over the range *h*_0_ ∈ [0, 0.2]. The two values used for *w*_*opt*_ are 1524 and 7616 (Table 2 in S1 File). We further use *μ* = 0.07 day^−1^.
(EPS)

S1 File Supporting text. (PDF)
