# Supplementary material for: Energetic costs of cellular and therapeutic control of stochastic mitochondrial DNA populations
Source: PLoS Comput Biol. 2019 Jun 26;15(6):e1007023. doi: 10.1371/journal.pcbi.1007023 (PMC6615642; doi:10.1371/journal.pcbi.1007023)
Supplement: S1 File — (PDF) [file pcbi.1007023.s015.pdf]

Supplementary Information

1 System Size Expansion

Stochastic birth-death processes can be described analytically by a master equation. For our model, with birth rate $\lambda(w, m)$ and constant death rate μ, we obtain:

$$\begin{aligned}\frac{\partial P_{i,j}}{\partial t} &= \lambda(i-1,j)(i-1)P_{i-1,j} + \mu(i+1)P_{i+1,j} \\ &+ \lambda(i,j-1)(j-1)P_{i,j-1} + \mu(j+1)P_{i,j+1} \\ &- (i+j)\Big(\lambda(i,j) + \mu\Big)P_{i,j} \end{aligned} \tag{1}$$

where $P_{i,j}(t)$ gives the probability of being in state $(i, j) = (w, m)$ (the time dependence is dropped for convenience). In general, this equation will not be analytically solvable and suitable approximation methods are required.

In this section, we use van Kampen's system size expansion (SSE) [1] to approximate the above equation. First, we derive the leading order solution of the expansion, known as the linear noise approximation (LNA). As we will see, the LNA provides useful insights and can accurately describe the dynamics of our system for short time-scales. However, for longer time-scales higher order terms are required to maintain accuracy.

Generally, a master equation can be written in the form

$$\dot{p_{\vec{n}}}(t) = \Omega \sum_{j=1}^{R} \left(\prod_{i=1}^{N} \mathbb{E}^{-S_{ij}} - 1 \right) f_j(\vec{n}, \Omega) p_{\vec{n}}(t) \tag{2}$$

where Ω is the system volume, R is the number of reactions involved, N is the number of species, $(S)_{ij}$ is the stoichiometry matrix, $\vec{n} = (n_1, n_2, \cdots, n_N)$ gives the number of particles of each species, $\mathbb{E}$ is a raising and lowering operator (its effect on an arbitrary function $f(\vec{n})$ is given by e.g. $\mathbb{E}_i f(\vec{n}) = f(\vec{n}+e_i)$ and $\mathbb{E}_i^{-1} f(\vec{n}) = f(\vec{n} - e_i)$ where e_i is a column vector with all zeros and a 1 at entry i), and $p_{\vec{n}}(t)$ is the probability distribution of states $\vec{n}$ at time t. The equations $f_j(\vec{n}, \Omega)$ specify the reaction rates. The SSE provides an expansion of this master equation in powers of the inverse square root of the system volume Ω.

The expansion starts by expressing the state of the system in terms of a deterministic and stochastic component. Consider a single species whose concentration and copy number are denoted by $\phi(t)$ and n, respectively. One expects the distribution of n to be centered around $\Omega\phi(t)$ and to have a width of order $\sqrt{n} \propto \sqrt{\Omega}$. This motivates the following equation

$$n_i = \Omega\phi_i(t) + \Omega^{1/2}\xi_i \tag{3}$$

where ξ_i describe the fluctuations around the deterministic solution $\phi_i(t)$. All terms in the master equation can now be expressed in the new fluctuation variables ξ_i according to the following transformations:

$$\begin{aligned} p_{\vec{n}}(t) &= \Pi(\vec{\xi}, t) \\ \mathbb{E}_i &\to 1 + \Omega^{-1/2}\frac{\partial}{\partial \xi_i} + \frac{1}{2}\Omega^{-1}\frac{\partial^2}{\partial \xi_i^2} + \cdots \\ \dot{p_{\vec{n}}} &= \frac{\partial \Pi(\vec{\xi}, t)}{\partial t} - \Omega^{1/2}\sum_{i=1}^{N}\frac{\partial \phi_i}{\partial t}\frac{\partial \Pi(\vec{\xi}, t)}{\partial \xi_i} \quad \text{(chain rule)} \\ f(\frac{n_i}{\Omega}) &= f(\phi_i + \Omega^{-1/2}\xi_i). \end{aligned} \tag{4}$$

Substituting Eqs. (4) into Eq. (2) gives

$$\begin{aligned} \frac{\partial \Pi(\vec{\xi}, t)}{\partial t} - \Omega^{1/2}\sum_{i=1}^{N}\frac{d\phi_i}{dt}\frac{\partial \Pi(\vec{\xi}, t)}{\partial \xi_i} = \Omega\sum_{j=1}^{R}\Bigg[&- \Omega^{-1/2}\sum_{i=1}^{N} S_{ij}\frac{\partial}{\partial \xi_i} + \frac{1}{2}\Omega^{-1}\sum_{i,k} S_{ij}S_{kj}\frac{\partial^2}{\partial \xi_i \partial \xi_k} \\ &+ O(\Omega^{-3/2})\Bigg] \\ &\times \left[f_j(\vec{\phi}) + \Omega^{-1/2}\sum_{i}\frac{\partial f_j(\vec{\phi})}{\partial \phi_i}\xi_i + O(\Omega^{-1})\right]\Pi(\vec{\xi}, t) \end{aligned} \tag{5}$$

which can be solved by collecting powers of $\Omega^{-1/2}$. Terms proportional to $\Omega^{1/2}$ form the macroscopic rate equations. The terms of order Ω^0 form a Fokker-Planck equation, the solution of which is the LNA:

$$\frac{\partial \Pi(\vec{\xi},t)}{\partial t} = -\sum_{i,j=1}^{2} A_{ij} \frac{\partial(\xi_j \Pi(\vec{\xi},t))}{\partial \xi_i} + \frac{1}{2}\sum_{i,j=1}^{2} B_{ij} \frac{\partial^2 \Pi(\vec{\xi},t)}{\partial \xi_i \partial \xi_j} \tag{6}$$

The coefficients A_{ij} and B_{ij} can be found by expanding Eq. (5) and are defined as

$$\begin{aligned} A_{ij} &= \sum_{k=1}^{R} S_{ik} \frac{\partial f_k}{\partial \phi_j} \\ B_{ij} &= \sum_{k=1}^{R} S_{ik} S_{jk} f_k \end{aligned} \tag{7}$$

The system we consider has two species ($N = 2$), four rate equations ($R = 4$) given by

$$\begin{aligned} f_1 &= w\lambda(w,m) \\ f_2 &= m\lambda(w,m) \\ f_3 &= w\mu \\ f_4 &= m\mu, \end{aligned} \tag{8}$$

and a stoichiometry matrix by

$$S = \begin{pmatrix} 1 & 0 & -1 & 0 \\ 0 & 1 & 0 & -1 \end{pmatrix} \tag{9}$$

where the two rows refer to the wildtype and mutant species, respectively, and the columns represent the reactions f_i.

In order to continue, it is useful to rewrite Eq. (5) as an expansion in derivatives with respect to $\vec{\xi}$, namely $\partial_t \Pi = \sum_i \left(-\frac{\partial}{\partial \xi_i} A_i + \frac{1}{2!}\frac{\partial^2}{\partial \xi_i^2} B_i - \frac{1}{3!}\frac{\partial^3}{\partial \xi_i^3} C_i + \cdots \right)$ where $A_i, B_i, \ldots$ are functions of $\vec{\xi}$, Ω and $\vec{f}$. Evolution equations for the moments $\langle \xi_i \xi_j \ldots \rangle$ can be obtained by multiplying this equation by $\xi_i \xi_j \ldots$ and integrating over all $\vec{\xi}$. Using integration by parts, the following set of coupled ODEs can be obtained for our $N = 2$ system:

$$\begin{aligned} d_t \langle \xi_1^2 \rangle &= 2A_{11}\langle \xi_1^2 \rangle + 2A_{12}\langle \xi_1 \xi_2 \rangle + B_{11} \\ d_t \langle \xi_2^2 \rangle &= 2A_{22}\langle \xi_2^2 \rangle + 2A_{21}\langle \xi_1 \xi_2 \rangle + B_{22} \\ d_t \langle \xi_1 \xi_2 \rangle &= (A_{11} + A_{22})\langle \xi_1 \xi_2 \rangle + A_{12}\langle \xi_2^2 \rangle + A_{21}\langle \xi_1^2 \rangle + B_{12} \end{aligned} \tag{10}$$

which can be solved to give

$$\begin{aligned} \langle \xi_1^2 \rangle = \frac{\mu w_{ss}^2}{(\partial_m \lambda m_{ss} + \partial_w \lambda w_{ss})^3} \Big(& 2m_{ss}(1 + \frac{m_{ss}}{w_{ss}})(\partial_m \lambda)^2 (\partial_m \lambda m_{ss} + \partial_w \lambda w_{ss}) t \\ & - 4(\partial_m \lambda)(\partial_w \lambda) m_{ss} - (\partial_w \lambda)^2 w_{ss} + 3(\partial_m \lambda)^2 m_{ss} \\ & + 4e^{(\partial_m \lambda m_{ss} + \partial_w \lambda w_{ss})t} \left\{ \partial_m \lambda \partial_w \lambda m_{ss} - (\partial_m \lambda)^2 m_{ss} \right\} \\ & + e^{2(\partial_m \lambda m_{ss} + \partial_w \lambda w_{ss})t} \left\{ (\partial_w \lambda)^2 w_{ss} + (\partial_m \lambda)^2 m_{ss} \right\} \Big) \end{aligned} \tag{11}$$

$$\begin{aligned} \langle \xi_2^2 \rangle = \frac{\mu m_{ss}^2}{(\partial_m \lambda m_{ss} + \partial_w \lambda w_{ss})^3} \Big(& 2w_{ss}(1 + \frac{w_{ss}}{m_{ss}})(\partial_w \lambda)^2 (\partial_m \lambda m_{ss} + \partial_w \lambda w_{ss}) t \\ & - 4(\partial_m \lambda)(\partial_w \lambda) w_{ss} - (\partial_m \lambda)^2 m_{ss} + 3(\partial_w \lambda)^2 w_{ss} \\ & + 4e^{(\partial_m \lambda m_{ss} + \partial_w \lambda w_{ss})t} \left\{ \partial_m \lambda \partial_w \lambda w_{ss} - (\partial_w \lambda)^2 w_{ss} \right\} \\ & + e^{2(\partial_m \lambda m_{ss} + \partial_w \lambda w_{ss})t} \left\{ (\partial_w \lambda)^2 w_{ss} + (\partial_m \lambda)^2 m_{ss} \right\} \Big) \end{aligned} \tag{12}$$

$$\begin{aligned} \langle \xi_1 \xi_2 \rangle = \frac{\mu w_{ss} m_{ss}}{(\partial_m \lambda m_{ss} + \partial_w \lambda w_{ss})^3} \Big(& -2(w_{ss} + m_{ss})(\partial_m \lambda)(\partial_w \lambda)(\partial_m \lambda m_{ss} + \partial_w \lambda w_{ss}) t \\ & + (\partial_w \lambda)^2 w_{ss} + (\partial_m \lambda)^2 m_{ss} - 2(\partial_w \lambda)(\partial_m \lambda)(w_{ss} + m_{ss}) \\ & + 2e^{(\partial_m \lambda m_{ss} + \partial_w \lambda w_{ss})t} \left\{ (\partial_m \lambda)(\partial_w \lambda)(w_{ss} + m_{ss}) - (\partial_w \lambda)^2 w_{ss} - (\partial_m \lambda)^2 m_{ss} \right\} \\ & + e^{2(\partial_m \lambda m_{ss} + \partial_w \lambda w_{ss})t} \left\{ (\partial_w \lambda)^2 w_{ss} + (\partial_m \lambda)^2 m_{ss} \right\} \Big) \end{aligned} \tag{13}$$

where $\langle\xi_1^2\rangle$, $\langle\xi_2^2\rangle$ and $\langle\xi_1\xi_2\rangle$ denote the wildtype and mutant variance and covariance, respectively. We used initial conditions with zero variance, assuming all cells start in exactly the same state. This case is the most general instance of our model. Under a linear noise approximation and for uncoupled cells, the behaviour of an arbitrary initial distribution of heteroplasmies can then be recovered by taking an appropriately weighted sum of the individual results for specific heteroplasmies starting at zero variance. Mutant and wildtype copy numbers are initialized at their steady state values. The means of w and m are calculated from the macroscopic rate equations at order $\Omega^{1/2}$ and are identical to the deterministic means. The intermediate-time forms of these expressions (when the exponentially decaying terms have died out) are given in Table 1 in the main text.

Heteroplasmy variance can be derived using a Taylor expansion which, keeping only the leading order, gives: $\mathrm{var}(h) \approx (\frac{\partial h}{\partial w})^2\sigma_w^2 + (\frac{\partial h}{\partial m})^2\sigma_m^2 + 2(\frac{\partial h}{\partial w})(\frac{\partial h}{\partial m})\mathrm{cov}(w,m) = \frac{1}{(w+m)^4}(m^2\sigma_w^2 + w^2\sigma_m^2 - 2w\,m\,\mathrm{cov}(w,m))$.

	$\lambda(w)$	$\lambda(m)$	$\lambda(w,m)$ (including $\lambda(w+m)$)
$\sigma_w^2(t)$	$\frac{\mu}{\partial_w\lambda}\left(e^{2w_{ss}\partial_w\lambda t}-1\right)$	$2\frac{w_{ss}}{m_{ss}}\mu(w_{ss}+m_{ss})t+\cdots$	$\frac{2\mu m_{ss}w_{ss}(w_{ss}+m_{ss})}{(m_{ss}\partial_m\lambda+w_{ss}\partial_w\lambda)^2}(\partial_m\lambda)^2\,t+\cdots$
$\sigma_m^2(t)$	$2\frac{m_{ss}}{w_{ss}}\mu(w_{ss}+m_{ss})t+\cdots$	$\frac{\mu}{\partial_m\lambda}\left(e^{2m_{ss}\partial_m\lambda t}-1\right)$	$\frac{2\mu m_{ss}w_{ss}(w_{ss}+m_{ss})}{(m_{ss}\partial_m\lambda+w_{ss}\partial_w\lambda)^2}(\partial_w\lambda)^2\,t+\cdots$
$\sigma_h^2(t)$	$\frac{2m_{ss}w_{ss}\mu}{(m_{ss}+w_{ss})^3}t$		

Table 1: **Analytical expressions for the means and variances according to the linear noise approximation.** Solutions are shown for wildtype, mutant, and heteroplasmy variances for various types of control. Dots indicate constant or exponentially decaying terms; full solutions are provided in Eqs. (11) - (13). Note that the initial rate of increase of heteroplasmy variance only depends on mtDNA copy number and turnover (see also [2]).

2 Feedback control of a heteroplasmic mtDNA population

2.1 Steady states of a linear feedback control

When mutant and wildtype mtDNA molecules have identical replication ($\lambda_m = \lambda_w$) and degradation ($\mu_m = \mu_w$) rates, infinitely many steady states exist (i.e. states in which $\lambda = \mu$). These steady states form lines in (w,m) space which can be straight (linear feedback control, Fig 1A), form segments of ellipses (quadratic feedback control) or take more complicated forms. Deterministic trajectories will asymptotically approach the steady state line while stochastic trajectories can fluctuate along the line thereby changing heteroplasmy. Similar lines of steady states are present in the relaxed replication model [3, 4].

A linear feedback control $\lambda(w,m) = c_0 - c_1(w+\delta m)$ (with $c_0, c_1 > 0$ constants) gives rise to a straight line of steady states, the slope of which is determined by δ. A small δ means that the steady state line intersects the mutant axis at higher copy number than the wildtype axis, meaning that total copy numbers are higher at $h=1$ than at $h=0$. In the extreme case of $\delta = 0$, mutant copy numbers can fluctuate off to infinity, though in practice their numbers will be bounded by space restrictions and resource competition. When $\delta > 1$ copy numbers will decrease as h increases; mutants are now sensed more than wildtypes, which could be caused by e.g. excessive production of reactive oxygen species by mutants (which is then sensed by the cell and incorporated in its feedback function).

Fig. 1 in the main text shows the near-equivalence of mean wildtype, mutant, and heteroplasmy dynamics between three different forms of control, all of the form $\lambda(w,m) = \lambda(w+\delta m)$: i) $c_0 - c_1(w+\delta m)$, ii) $d_0 + d_1/(w+\delta m)$ and iii) $s_0 - s_1(w+\delta m)^2$ with $c_0, c_1, d_0, d_1, s_0, s_1 > 0$. Fig. 1B, C, D shows that variance dynamics are also nearly identical.

We merely show that these different controls *can* give rise to similar dynamics by parameterising them such that they do. First, we set the variances of each control to be equal in the absence of mutants. From Table 1 it can be seen that the wildtype variance is now completely specified by the mtDNA degradation rate μ, the steady state copy number w_{ss} and the control derivative $\partial_w\lambda(w)$ evaluated at steady state. The latter quantity is given by i) $-c_1$, ii) $-d_1/w_{ss}^2$ and iii) $-2s_1w_{ss}$ for the three controls defined above. These expressions were all set to be -7.21×10^{-6}, a value that was chosen such that the wildtype steady state distribution has a coefficient of variation of $CV = 0.1$. This value is arbitrary, other choices of give similar results. We use a deterministic wildtype steady state of 1000 (in the absence of mutants), which fixes the other control parameters. Note that it is not surprising that these different controls yield similar dynamics after we have parameterised them to do so; we want to illustrate these similarities to stress that *which quantity is being controlled can be more important than how it is controlled.*

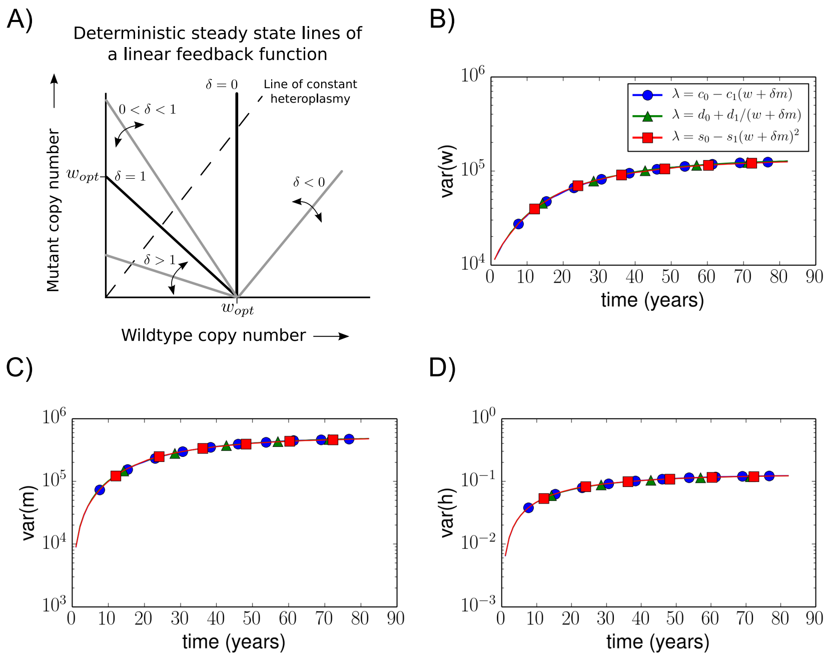

Figure 1: **A linear feedback control has straight steady state lines.** The deterministic steady state lines of the feedback control given in Eq. (4) in the main text, using our linear version of $\lambda(w, m)$, are shown in (w, m) space for various values of δ (grey lines show particular examples of ranges of δ). Constant heteroplasmy lines form straight lines through the origin. **B, C, D) Equal variances for different feedback control mechanisms.** Three different controls (see legend), all of the form $\lambda(w + \delta m)$ with $\delta = 0.5$, show nearly identical wildtype, mutant and heteroplasmy variances. Other parameters used are $N_{ss} = 1000$ (referring to the steady state copy number present in the absence of mutants), $\mu = 0.07$ (corresponding to a half-life of 10 days), and initial copy numbers $(w_0, m_0) = (920, 160)$ (corresponding to an initial heteroplasmy of ~ 0.15).

2.2 Homogenate heteroplasmy values

As mentioned in the main text, without explicit selection for either mtDNA species, mean cellular heteroplasmy remains constant at its original value, i.e. $\frac{m_0}{m_0+w_0}$ where w_0 and m_0 denote the initial wildtype and mutant copy numbers, respectively. For long times, the fraction of cells that fixate on mutant species (i.e. $h = 1$) is given by $m_0/(w_0 + m_0)$ and these cells have mean copy number w_{opt}/δ, whereas wildtype cells have mean copy number w_{opt}. We can now calculate the homogenate heteroplasmy at long times (i.e. times at which all cells have fixated) as

$$\langle h \rangle_{homog,\, t\to\infty} = \frac{m_0}{m_0 + \delta w_0}. \tag{14}$$

We note that Eq. 14 only holds when $\delta > 0$.

3 The relationship between resource consumption and energy production: $s(r_i)$

3.1 A linear output model

In our cost function we need to specify how s, the power supply measured in ATP/s (including leak) of a mitochondrion, depends on r_i, a quantity resembling the resource consumption rate of a mitochondrion or type i (w or m). For robustness, we use two different equations for $s(r_i)$.

The first model is based on measurements in isolated mitochondria which found a linear relationship between r_i and s (e.g. [5, 6, 7]), with r_i referring to oxygen consumption rate. Specifically, we use the following equation:

$$s(r_w) = \phi(r_w - \beta) \tag{15}$$

where ϕ can be mapped to the effective P/O ratio (explained in section 4.1) and β indicates the respiration rate at zero energy production and therefore specifies the amount of leak. We used the data from Ref. [5] to fit the parameters of this linear model (Table 2, section 4.1), the result of which is shown in Fig 2A in section 5.

It is not clear, however, whether the observations made in isolated mitochondria, without any interactions between the mitochondria and the nucleus or the endoplasmic reticulum, still hold *in vivo*. This is one of the reasons to also consider another type of model, as described below.

3.2 A saturating output model

Our cost function contains a term which penalises the consumption of resources, meaning that the cheapest state is the one that satisfies demand with the smallest net resource consumption rate. A linear function $s(r_i)$ then implies that, for a given demand, the optimal number of mitochondria is the minimum number required to satisfy the demand with these mitochondria respiring as fast as they can (as can be seen in Fig 2E). However, containing a minimally required number of mitochondria will make a cell less robust to stochastic fluctuations in both mitochondrial copy numbers and demand. Moreover, mitochondria are known to have large spare capacities [8, 9] indicating that in resting state they do not operate near their limits. We therefore expect that there is some extra cellular cost associated with this 'maximally respiring' state, causing it to be non-optimal in resting conditions.

This is why we have chosen to use a second model which describes a saturating relationship between r_i and s (visualised in Fig 2A). Note that we do not claim that mitochondria become less efficient as they respire faster, we impose the saturating shape merely to effectively assign a higher cost to high respiring states. By imposing this saturation, the variable on the y-axis of Fig 2A can be interpreted an 'effective energy production'. We will refer to the two models as 'the linear output model' and 'the saturating output model'.

A changing energy production efficiency is not entirely unreasonable, though, because in experiments with isolated mitochondria one usually uses a particular substrate (or a particular combination of substrates) whereas a larger mixture of substrates will be available in the cell, and the relative presence of each substrate may fluctuate over time. The efficiency of respiration depends on the kind of substrate that is used, so it may be possible that at high demand (and high respiration) the substrate of first choice has become limited and another less efficient substrate is used instead. Also, it was suggested that spare capacity can be caused by an increase in substrate entrance in the tricarboxylic acid (TCA) cycle [10]. This would mean that a state of high respiration is caused by an increase in electron transport chain substrates (e.g. NADH and $FADH_2$). A 'push' to the proton pumping complexes instead of a 'pull' at the ATP synthase would lead to an increase in the electrochemical gradient across the membrane and therefore an increase in leak. These arguments are speculative but show that the saturation model may not be unreasonable.

The saturating model is defined by the equation:

$$s(r_w) = 2\frac{s_{max}}{1+e^{-kr_w}} - (1+\Delta)s_{max}$$

$$s(r_w) = 2\frac{s_{max}}{1+e^{-kr_w}} - 1.1s_{max} \tag{16}$$

where k is the rate at which the model saturates, s_{max} is an indication of the maximum energy production rate, and $\Delta > 0$ is introduced to ensure that, like in the linear model, a nonzero amount of resource is required to maintain a zero energy output (due to proton leak). As proton leak accounts for $\sim 10\%$ of the total energy production in the linear model, we chose $\Delta = 0.1$. We will set the parameter values k and s_{max} such that the saturating and linear model show similar behaviour for low values of r_w (section 4.5), as shown in Fig 2A of section 5.

4 Parameter values for the cost function

Some of our results will be a consequence of the exact structure of our cost function, and might have been different if another type of cost function was used. We would argue, however, that the main elements in our cost function are quite general: terms involving supply, demand, and resource. We aimed at making our cost function simple, and using biologically interpretable parameters. We do not aim to give a detailed kinetic description of the energetic costs involved, but present a simpler description that allows us to compare distinct strategies relative to each other rather than providing absolute costs. We use our cost function as a tool to characterise cost landscapes and begin to explore optimal control strategies.

In the spirit of 'back-of-the-envelope' reasoning in biology [11] we seek plausible and interpretable parameter estimates, using both order-of-magnitude estimations and values found in the literature. Default parameter values are summarised in Table 2.

The final goal is to obtain a cost for a state with a certain number of mtDNA molecules; we therefore need to express our cost as a cost 'per mtDNA molecule'. Because the density of mtDNA molecules

within the mitochondrial network seems to be roughly constant [12], we assume every mtDNA molecule is associated to a particular amount of mitochondrial volume which we here define as a 'mitochondrial unit'. All our parameters refer to these mitochondrial units.

4.1 Mitochondrial energy production and leak: ϕ and β

Mitochondrial respiration is a process by which energy from nutrients is converted into (amongst others) ATP. Part of this process involves the pumping of protons across the inner mitochondrial membrane to create an electrochemical potential across the membrane. Energy is released when protons flow back into the matrix and this energy can be used to create ATP. However, the coupling between proton pumping and ATP synthesis is not perfect and protons can leak through the membrane, reducing the efficiency of respiration. An often measured quantity in experimental studies is the mechanistic P/O ratio [13, 14] which refers to the theoretical maximum amount of ATP (P) produced per oxygen (O) reduced by the respiratory chain. The effective P/O ratio is more physiologically relevant and takes into account leak [7].

In our linear model we assume a linear relationship between mitochondrial ATP production rate and mitochondrial oxygen consumption rate, based on experimental data [5, 7, 6]. In Refs. [5, 6] measurements show an almost perfect linear relationship between these two quantities, which is consistent with data from Ref. [7]. The parameters ϕ and β correspond to the slope and intercept of the linear function, respectively. In Ref. [5] this slope was measured to be 2.03 ± 0.13 for isolated pectoralis muscle cell mitochondria in the presence of pyruvate and malate; we have decided to use $\phi = 2$, mainly based on these experiments.

The value of β is an indication of the 'leakiness' of the mitochondrion: it represents the rate of oxygen consumption that is required to balance the leakage of protons across the membrane in order to maintain the mitochondrial membrane potential. To obtain a consistent value for β we also use the data presented in Ref. [5]. Their measurements find that β is about a tenth of the maximum respiration rate. This maximum respiration rate is obtained by adding high (unlimited) concentrations of ADP; the state of the cell in these conditions is known as state 3_{ADP}. Because state 3_{ADP} does not necessarily correspond to *in vivo* conditions, we define the respiration rate in this state as $r_{max,t}$: the maximum 'theoretical' respiration rate. We will fix $r_{max,t} = 1$ and use this to scale our other parameters. This means that our parameter value for β is $\beta = 0.1$.

We stress that though we have based our parameter values here on a specific study, changes in their values will not affect the qualitative structure of our cost function but merely changes the slope and intercept of the linear output function defined in Eq. (15).

4.2 Resource and supply and maintenance cost: r_{max}, r_n and s_n

A cell *in vivo* is unlikely to experience the high concentrations of ADP present in state 3_{ADP} (defined above). We therefore set our parameter r_{max}, the physiological maximum respiration rate, to be slightly below $r_{max,t}$, i.e. $r_{max} = 0.95 \cdot r_{max,t} = 0.95$.

We introduce r_n and s_n as the 'normal' respiration rate and ATP production rate which are present in resting conditions, respectively. Their values are not used directly in our model equations, but only to connect our parameter estimates to actual physiological values (section 4.8). Mitochondria have spare capacity, i.e. in normal unstressed conditions they use only part of their maximal oxygen consumption rate (OCR). The amount of spare capacity is usually measured as the fold-change in OCR that occurs after adding FCCP to cells, a mitochondrial uncoupler. Several measurements of the fold-change in OCR are: in the range (2-4)-fold [15], 1.4- to 2.5-fold [10], about 2-fold [8] and about 2.5-fold [9]. The maximum respiration rate when adding FCCP is known as State 3_{FCCP}, and is higher than State 3_{ADP} (see e.g. [16]). We interpret the spare capacity as the ratio State $3_{\text{FCCP}}/r_n$, meaning that the ratio r_{max}/r_n is lower than this. We have decided to take $r_{max}/r_n = 1.5$, meaning that $r_n = r_{max}/1.5 \approx 0.63$. For the linear output model, the value of s_n is now simply $s_n = s(r_n) \approx 1.1$ (this abstract value is related to actual values in ATP/s in section 4.8).

4.3 Maintaining, building and degrading: ρ_1, ρ_2 and ρ_3

The model organism with the most quantitative data on mitochondrial energy budgets is budding yeast, *Saccharomyces cerevisiae.* Reasoning that the scales of biophysical costs of mitochondria are likely comparable across eukaryotes, we first draw from this literature to motivate order-of-magnitude estimates for a range of essential mitochondrial processes. We will later construct parallel estimates using other organisms.

We first focus on estimating the mitochondrial building cost ρ_2 (in ATP). We provide three distinct estimations and combine them to obtain our final estimate.

For the first estimation we use a list of mitochondrial proteins in yeast [17], and obtain information on turnover rates, abundance (per yeast cell), and lengths (amino acid length) of these proteins by using the Saccharomyces Genome Database [18]. We end up with a list of ~ 200 mitochondrial proteins in yeast *S. cerevisiae.* We incorporate the observation that it takes about 5.2 ATP molecules to elongate a growing peptide chain by adding an amino acid [19], which means that the total synthesis cost of the mitochondrial proteins included in our list is given by $5.2\sum_i \text{length}_i\text{abundance}_i \approx 2 \times 10^{10}$ ATP per yeast cell. The known number of mitochondrial proteins in *S. cerevisiae* is on the order of 1000 [20]; we will therefore assume that the protein synthesis cost obtained from our protein list corresponds to roughly a fifth of the total mitochondrial protein synthesis cost. We might expect that the proteins best known [17] are the more abundant ones, meaning that our final cost is likely to be an overestimate. We also assume that all of the mitochondrially associated proteins are used exclusively for mitochondrial function. In *E. coli*, the mitochondrial protein synthesis cost represents ∼50% of the total mitochondrial synthesis cost (two other major contributors are phospholipid synthesis and RNA synthesis)[19]; we will make the assumption that this observation in *E. coli* holds in mitochondria as well. This brings the total mitochondrial building cost in a single yeast cell to be $\sim 1.9 \times 10^{11}$ ATP. Assuming 50-100 mtDNA molecules per yeast *S. cerevisiae* cell [21], the building cost associated with a single mtDNA molecule (and therefore the building cost of a mitochondrial unit) is given by $(2 - 4) \times 10^9$ ATP.

For our second estimation we use the total protein weight of a single mitochondrion which was measured to be about 3×10^{-10} mg in rat liver [22], as well as the typical weight of a single protein which is about 5×10^{-17} mg [19]. This means that a mitochondrion contains about 6×10^6 proteins. Using that the typical length of a protein is 300 amino acids [23, 24, 19] together with the 5.2 ATP cost of adding amino acids and the estimation that protein costs represent 50% of the entire building cost, the mitochondrial building cost is estimated to be 2×10^{10} ATP. Note that this cost does not necessarily represent our mitochondrial unit because it is unknown how many mtDNAs a 'mitochondrion' corresponded to when measuring its weight in Ref. [22].

The third estimation is based on the building cost of an *E. coli*, which is about 10^{10} ATP [19]. Keeping in mind that we want the building cost of a fraction of mitochondrial volume corresponding to a single mtDNA molecule, we need to convert the building cost of an *E. coli* (which has a volume of about 1 μm^3) to represent our mitochondrial unit. It was estimated that the total mitochondrial network length in yeast *S. cerevisiae* is about 25 μm with a total mtDNA copy number of 50-100 [21]. Assuming that the mitochondria form tubules with a constant diameter of 300 nm [25] gives a total mitochondrial volume of about 1.8 μm^3; another total mitochondrial volume estimate in yeast *S. cerevisiae* is 1.5 μm^3 [25]. Uniform distributions for the mitochondrial volume (1.5-1.8 μm^3) and mtDNA copy numbers (50-100) leads to a volume of $(2.3 \pm 0.5) \times 10^{-2}\mu\text{m}^3$ per mitochondrial unit. This means that rescaling the *E. coli* building cost gives us an estimate of $(2.3 \pm 0.5) \times 10^{-2} \cdot 10^{10} = (2.3 \pm 0.5) \times 10^8$ ATP to build a single mitochondrial unit. Note that this may represent an overestimation because *E. coli* is a unicellular organism, whereas the mitochondrion is an organelle which cannot survive in isolation [26].

While there are differences in these estimates, arising both from uncertainty and different quantitative lines of reasoning, they together give an overall scale for mitochondrial building cost of around 10^9 ATP. Because in our model we only need a rough estimate of the mitochondrial building cost, we use $\rho_2 = 10^9$ ATP.

We interpret the maintenance cost, denoted by ρ_1, as the cost in molecules ATP/s corresponding to, for example, maintaining the mitochondrial lipid membranes, importing/exporting proteins, and synthesizing new proteins. To obtain an estimation of ρ_1, we again use the Saccharomyces Genome Database [18]. We can calculate the cost of continuously turning over the ~ 200 proteins in our list (obtained from [17]), leading to $5.2\sum_i \text{length}_i\text{abundance}_i(\text{degradation rate})_i \approx 6 \times 10^5$ ATP/s per yeast cell. In other words, the maintenance cost per second of our set of mitochondrial proteins is about five orders of magnitude less than their synthesis cost. We therefore assume $\rho_1 = 10^{-5}\rho_2$.

The mitochondrial degradation cost ρ_3 is the most challenging parameter to estimate, as the process of mitochondrial degradation remains poorly characterised. Protein production and biosynthesis costs form the bulk of mitochondrial production requirements, and from cell-wide studies on energy budgets are among the most considerable demands in cell biology. We therefore assume that degradation has lower energy requirements than production, and set its upper limit at $\rho_3 = 0.1 \cdot \rho_2$. Lowering ρ_3 further has little impact on the outcomes of our model.

4.4 Demand and resource availability: D and R

We want to model two kinds of cells: low copy number cells (1000 wildtype mtDNAs in resting state) and high copy number cells (5000 wildtype mtDNAs in resting state). Denoting this desired number of

'normal' mitochondria by w_n (which are assumed to operate at the 'normal' production rate s_n (section 4.2)), we obtain

$$D = w_n(s_n - \rho_1 - \mu\rho_3 - \lambda\rho_2) \quad (17)$$

where μ and λ are the degradation and replication rates per second. This equation states that the overall net output of w_n mitochondria exactly satisfies demand. Using $w_n = 1000$ and $w_n = 5000$ leads to $D \approx 1055$ and $D \approx 5275$ ATP/s. These abstract values for demand are mapped to actual cellular ATP demands in section 4.8.

The parameter R denotes the maximum rate at which resource can be consumed by all of the mitochondria together and represents a cellular resource availability. In normal resting state the total resource that is consumed is $w_n r_n$, and the resource consumed when these mitochondria respire as fast as they can is $w_n r_{max}$. We then assume this maximal respiration rate is achieved by using all of the available resources, i.e. $R \approx w_n r_{max}$. It may be, however, that a state of maximum respiration can only be maintained for a short time, and in our cost function we want to describe the 'steady state cost' for different states. We therefore use $R < w_n r_{max}$ (R now denotes the maximal respiration rate that can be maintained for longer periods of time). We chose to use $R = 0.8 w_n r_{max}$ leading to $R = 760$ and $R = 3800$ for $w_n = 1000$ and $w_n = 5000$, respectively.

4.5 Saturating model parameters: s_{max} and k

We set the parameters of the saturating model described in Eq. (16) such that it matches the linear model for low respiration. This lead to $k = 3.0$ and $s_{max} = 1.54$.

4.6 The cost of resource consumption α

The value of α, i.e. the scaling parameter that appears in the cost function given by

$$C(w, m) = |D - S(w, m)| + \alpha(w r_w + m r_m) \quad (18)$$

is hard to determine. Its value describes the cost of a unit of resource consumption relative to the cost of a unit of 'energy deficiency' (the cost of $S(w, m)$ being one energy-unit below D). We estimated that a penalty for resource consumption usage should be about an order-of-magnitude less than the penalty for not-satisfying demand, and have therefore decided to assume $\alpha = 0.1$. We note that the value of α has no influence on the shape of the demand-satisfying region, it only changes the relative costs within (and outside of) the region.

4.7 Mutant parameters: ϵ_1, ϵ_2

In the main text we vary the parameter ϵ_1, describing the resource uptake rate of mutants relative to wildtypes. Additionaly, mutants can be less efficient than wildtypes, producing less energy per resource consumed; we denote this lower mutant efficiency by $\epsilon_2 \in [0, 1]$. Because the number of protons that are pumped across the mitochondrial inner membrane by the electron transport chain complexes for every unit of resource (NADH) that is consumed is fixed, a lower ϵ_2 would have to mean that either i) the mutation has increased proton leak (or other ways of depolarising the membrane), or ii) the mutation has made the ATP synthase dysfunctional. The value of ϵ_2 can be related to the P/O ratio of the mutants relative to that of the wildtypes. Most mtDNA mutations, however, affect the electron transport chain complexes themselves and are therefore likely to reduce the flow of resources through the chain (which would mean a low value for ϵ_1). This is why we assume $\epsilon_2 = 1$ in our main model and only vary the parameter $0 < \epsilon_1 < 1$. For completion, here in the SI we provide a heatmap showing the cost in (w, m) space for various values of ϵ_2 (Fig 3).

4.8 Parameter units

We can relate our parameter values to actual values, e.g. expressing our demand D in ATP/s. As an example of an ATP demand we use the ATP production rate in unstressed human skin fibroblasts. Assuming that these healthy cells satisfy their demand, their net ATP production rate should equal their ATP demand. The rate of ATP production in skin fibroblasts was estimated to be about 10^9 ATP/s, the large majority of which is supplied by mitochondria [27]. The number of mtDNAs in healthy human skin fibroblasts was measured to be roughly in the range 2400-5200 [28] (the variation in copy number was partly due to variation in ages of the individuals), and we will use the value 4000 as an estimation.

Using $w_n = 4000$ we obtain

$$
\begin{aligned}
10^9 &= 4000(s_n - \rho_1 - \lambda\rho_2 - \mu\rho_3) \\
&\approx 4000(s_n - \rho_2(10^{-5} + \lambda + 0.1\mu) \\
&\approx 4000\Big(s_n - 10^9\Big[10^{-5} + 0.07/(24 \cdot 3600) + 0.1 \cdot 0.07/(24 \cdot 3600)\Big]\Big)
\end{aligned}
\tag{19}
$$

Here we used an mtDNA half-life ($T_{1/2}$) of 10 days, giving a degradation rate $\ln(2)/10 \approx 0.07$ day^{-1}, and assumed that the cells are in steady state with $\lambda = \mu$. This leads to $s_n \approx 2.6 \times 10^5$ ATP/s meaning that, considering we used $s_n \approx 1.1$, our parameters s_n, D are expressed in units of about 2.6×10^5 ATP/s. This means that, in our units, the parameter values we use for ρ_1, ρ_2 and ρ_3 are $\rho_1 \approx 0.04$, $\rho_2 \approx 3828$, and $\rho_3 \approx 383$.

Par.	Description	Default value
D	Mitochondrial energy demand in ATP/s. The default values given are in units of 2.6×10^5 ATP/s, as explained in section 4.8. We introduce two values of D, one for high and one for low copy number cells.	5110.7 (high)<br>1022.1 (low)
R	Maximum rate of resource supplied by the cell per second to be used by the mitochondria. We use the term 'resource' as an amalgamation of different mitochondrial resources, e.g. NAD(H), pyruvate, lactate, succinate, ADP, P_i, and oxygen. Two different values for R are used, corresponding to high and low copy number cells.	3800 (high)<br>760 (low)
ϕ	ϕ is related to the effective P/O ratio of a wildtype mtDNA molecule, it represents the slope of the linear relationship between r_w and s given in Eq. (15).	2.0
β	β denotes the resource consumption rate (in 'resource' per second) at zero energy production, and is therefore a measure of proton leak. Because we fix the maximum resource consumption rate to be 1 (section 4.1), β provides the fraction of resource consumption that is destined for 'futile' leakage.	0.1
k	Parameter describing the saturation of the saturating model.	3
s_{max}	An indication of the aximum energy supplied by a wildtype mtDNA molecule in the linear model in ATP/s.	1.54
r_{max}	Maximum rate of resource uptake by an mtDNA molecule (i.e. by a mitochondrion containing the mtDNA molecule). This can be interpreted as the maximum flow through the respiratory chain.	0.95
ρ_1	The mitochondrial maintenance cost in units of 2.6×10^5 ATP/s.	0.04
ρ_2	The mitochondrial building cost in units of 2.6×10^5 ATP.	3828
ρ_3	The mitochondrial degradation cost in units of 2.6×10^5 ATP.	383
ϵ_1	The mutant energy production efficiency (the energy produced per unit of resource consumed) relative to that of wildtype mtDNA molecules. A low value of ϵ_1 can be caused by a high proton leak or a deficient ATP synthase.	free parameter $\epsilon_1 \in [0, 1]$
ϵ_2	The mutant resource uptake rate relative to that of wildtype mtDNA molecules. We will mainly use $\epsilon_1 = 1$ and $\epsilon_2 < 1$ because mtDNA mutations usually affect the proton pumping electron transport chain complexes. A defect in e.g. complex I will reduce its activity and therefore also the rate of consumption of NADH.	free parameter $\epsilon_2 \in [0, 1]$
α	Scaling parameter in the cost function in front of resource consumption term.	0.1
w_{opt}	The cheapest value for w when $m = 0$, using all of the above parameter values. We have four values for w_{opt}, corresponding to the linear and saturating output models at low and high copy number.	1524 (sat. low)<br>7616 (sat. high)<br>638 (lin. low)<br>3129 (lin. high)

Table 2: **Parameters used in our mitochondrial cost function with their descriptions.** Parameter values are derived and motivated in section 4. In this table, 'high' and 'low' refer to high and low copy numbers, respectively, and the abbreviations 'sat.' and 'lin.' are used to indicate our saturating and linear output models.

5 Cost function outputs

5.1 Output visualisations

Fig 2A shows the behaviour of Eqs. (15) - (16) for various parameter values. Note that none of the lines in the figure crosses the origin, because even when no ATP is created, respiration is required to maintain the gradient which would otherwise be lost due to proton leak. Fig 2B-E shows how resource consumption rate and cost change as the number of mtDNAs changes. Three regimes can be distinguished: 1) there are too few mitochondria present to satisfy demand and they use their maximum possible resource uptake to get their energy production rate as close to D as possible; 2) demand can be satisfied and the cost now only depends on the amount of resource that is used; 3) resource has become limiting and demand cannot be satisfied any more. The main difference between the two models is that in the linear model,

when demand is satisfied, the cheapest state is the one with the smallest mtDNA copy number possible whereas the saturating model is cheapest at a higher copy number. This is because mitochondria in the saturating model becomes more efficient as less resources are being consumed per mitochondrion.

Figs 3A,B show the cost function as a heatmap in (w, m) space. This figure is similar to Fig 2 in the main text except that here we have fixed $\epsilon_1 = 1$ and varied ϵ_2. Mutants are now less tolerated because they consume just as many resources as wildtypes, but still produce less output. It is now not the case that intermediate heteroplasmies are less efficient; *intermediate heteroplasmies are only less efficient when* $\epsilon_1 < 1$ *(mutants consume less resource) and when a saturating output model (Fig 2) is used.* When $\epsilon_1, \epsilon_2 < 1$, it is possible for intermediate heteroplasmies to be less efficient but the smaller the value of ϵ_2, the smaller the range of values of ϵ_1 for which this is true; therefore, the effect of intermediate heteroplasmies being less efficient will be most easily observed when the mutant efficiency is close to that of the wildtypes.

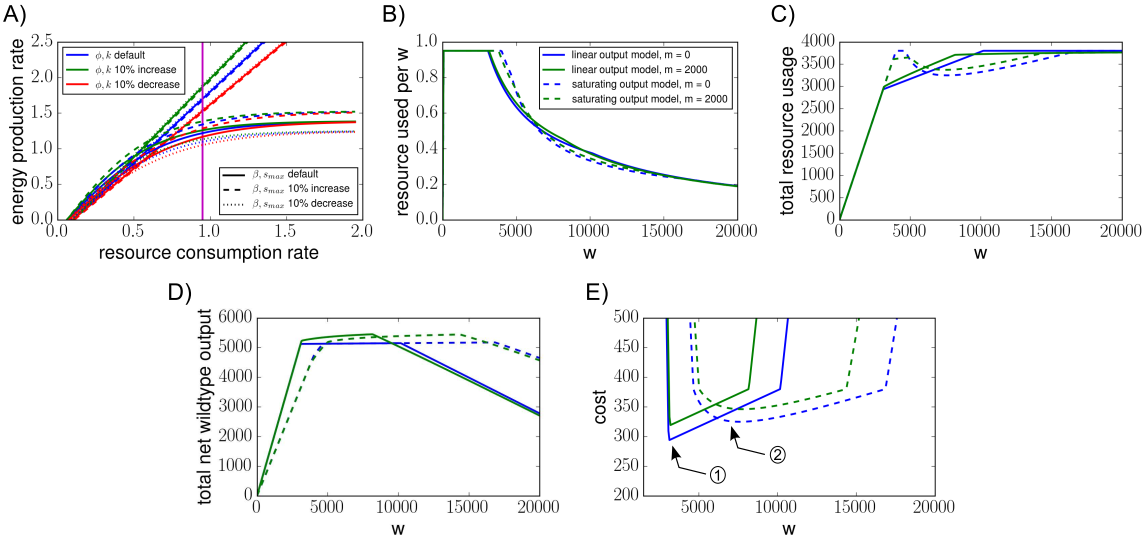

Figure 2: **Relationship between resource consumption and energy output. A)** The energy production rate of a single wildtype mitochondrion as a function of its resource consumption rate is shown, as given by Eqs. (15) and (16). For the linear model (corresponding to the straight lines) the parameters ϕ and β are changed by 10%, for the saturating model we vary s_{max} and k. The magenta line indicates r_{max}. **B)** As w increases, demand is shared between more mitochondria and each individual one can afford to consume resources at a lower rate (the same figure legend applies for figures C, D and E). **C)** The total resource consumption increases with w because the mitochondria need to consume a non-zero amount of resources to produce a net energy output and each mitochondrion comes with a maintenance cost. **D)** The total energy produced by wildtypes increases when mutants are present. **E)** When demand is satisfied, the cost increases with w in the linear model, resulting in minimal costs when copy numbers attain the minimum number required to satisfy demand (1). In contrast, for the saturating model the cost decreases at first because as individual resource consumption drops, the energy production efficiency increases. Minimum cost now occurs when mitochondria are working most efficiently (2). Parameters $\epsilon_1 = 0.1$ and $\epsilon_2 = 1.0$ were used.

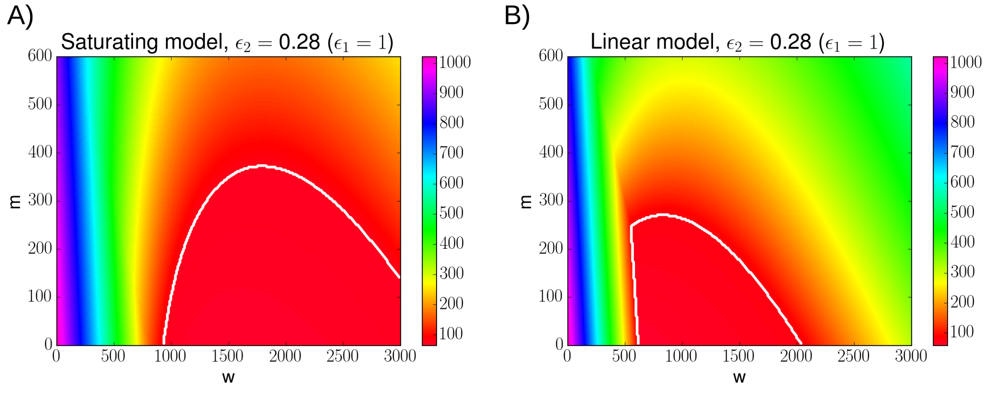

Figure 3: **Changing mutant efficiency (ϵ_2) does not lead to expensive intermediate heteroplasmies. A), B)** Similar to Fig 2 in the main text, these figures show the cost values in (w, m) space, but now as a function of ϵ_2 (mutant efficiency) instead of ϵ_1. This time we show the cost in the entire space. The white lines show the region in which demand is satisfied for our default parameter values. Because mutants consume the same amount of resource as wildtypes ($\epsilon_1 = 1$), resource becomes limiting at relatively low values of m compared to when $\epsilon_1 < 1$. Note that intermediate heteroplasmies are not less efficient here.

5.2 Expensive intermediate heteroplasmies - explanation and robustness

Here we attempt to provide an explanation for why, in certain cases, intermediate heteroplasmy values are more expensive than high or low values. In these cases, in high heteroplasmy regions it is more efficient to increase heteroplasmy even more, whereas in low h conditions it is more efficient to decrease h; this automatically implies there exists some intermediate heteroplasmy value that is least efficient. Figs 4A, B show the amount of resource consumed by individual wildtype and mutant mitochondria in four different example states. All states have identical total copy numbers ($w + m = 10^4$) and total outputs (equal to demand), but different heteroplasmy values ($h = 0.1, 0.3, 0.7$ and 0.9). When heteroplasmy increases, the individual resource consumption rates r_w and r_m both increase to compensate for the higher mutant copy number; this is true both in a low-h region (h increases from 0.1 to 0.3, Fig 4A) and a high-h region (h increases from 0.7 to 0.9, Fig 4B). However, the total resource consumption rate does not necessarily increase because the increase in h has caused a number of wildtype mitochondria to become mutants, thereby decreasing the combined wildtype resource usage. Computing the values of the resource consumption rates for the states with $h = 0.1$ and 0.3, while referring to Fig 4A, gives:

$$\begin{aligned} w_1 r_{w_1} &= 9000 \cdot r_{w_1} \approx 3227 \\ m_1 r_{m_1} &= 1000 \cdot r_{m_1} \approx 125 \\ w_2 r_{w_2} &= 7000 \cdot r_{w_2} \approx 2981 \\ m_2 r_{m_2} &= 3000 \cdot r_{m_2} \approx 447, \end{aligned} \tag{20}$$

while the states $h = 0.7$ and 0.9 (referring to Fig 4B) give

$$\begin{aligned} w_1 r_{w_1} &= 3000 \cdot r_{w_1} \approx 1982 \\ m_1 r_{m_1} &= 7000 \cdot r_{m_1} \approx 1618 \\ w_2 r_{w_2} &= 1000 \cdot r_{w_2} \approx 846 \\ m_2 r_{m_2} &= 9000 \cdot r_{m_2} \approx 2665. \end{aligned} \tag{21}$$

Comparing the states with $h = 0.1$ and $h = 0.3$, the *total* rates of resource usage are 3352 and 3428 respectively; the lower heteroplasmy state is more efficient. However, when heteroplasmies are higher, the high heteroplasmy state (0.9 rather than 0.7, with total resource usage 3511 vs 3600) is most efficient. This effect is due to the nonlinearity of Eq. 16.

Next, we investigate how robust the existence of an expensive intermediate heteroplasmy value is. Let h_{max} denote the value of h with maximum cost at fixed total copy number ($w + m$). Fig 5A shows h_{max} as a function of both total copy number and ϵ_1, setting all other parameters to their default values. The range of total copy numbers was chosen to correspond roughly to the range for which demand can be satisfied in a fully wildtype cell (with the objective of considering 'plausible' copy number regions). We see that an intermediate value of h_{max} exists for a wide range of parameter values, but only for large enough ϵ_1. This means we only expect to see expensive intermediate h values when neither of the two mtDNA species are severely dysfunctional. We further varied the parameters ρ_1 and s_{max} which play important roles in the cost function overall (ρ_1) and the specifics of the saturating output model (s_{max}),

examples of which are shown in Fig 5B,C,D which illustrates their values have little influence on h_{max}. Similar results were obtained by varying the parameters ρ_2 and ρ_3 by an order of magnitude.

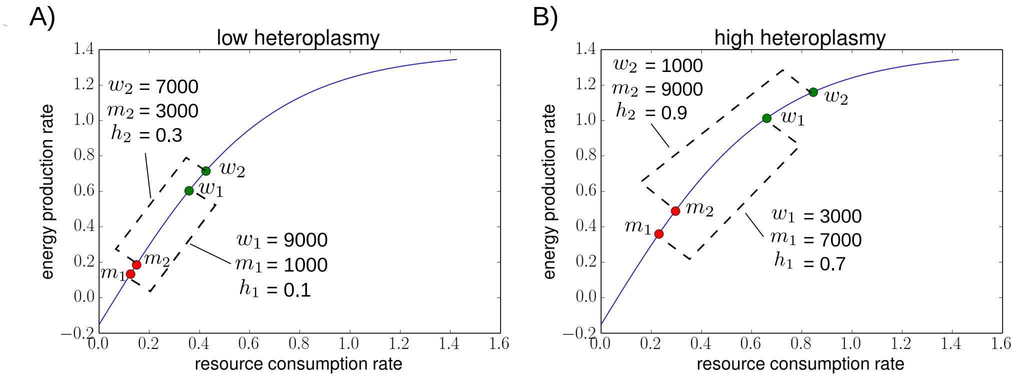

Figure 4: **Intermediate h values require more resources to satisfy demand, but only if mutants consume less resources. A)** The resource consumption rates and energy production rates of wildtypes and mutants are shown for two states: $(w_1, m_1, h_1) = (9000, 1000, 0.1)$ and $(w_2, m_2, h_2) = (7000, 3000, 0.3)$. In both cases, the total energy output is equal to the demand. When heteroplasmy is higher ($h = 0.3$), the individual resource consumption rates are higher in order to maintain a constant total energy output. Overall, the state with $h = 0.1$ uses the least resources (Eqs. 20). $\epsilon_1 = 0.35$ was used. **B)** This figure is similar to figure (D) but now the two states $(w_1, m_1, h_1) = (3000, 7000, 0.7)$ and $(w_2, m_2, h_2) = (1000, 9000, 0.9)$ are compared. The state with $h = 0.9$ uses the least resources (Eqs. (21)).

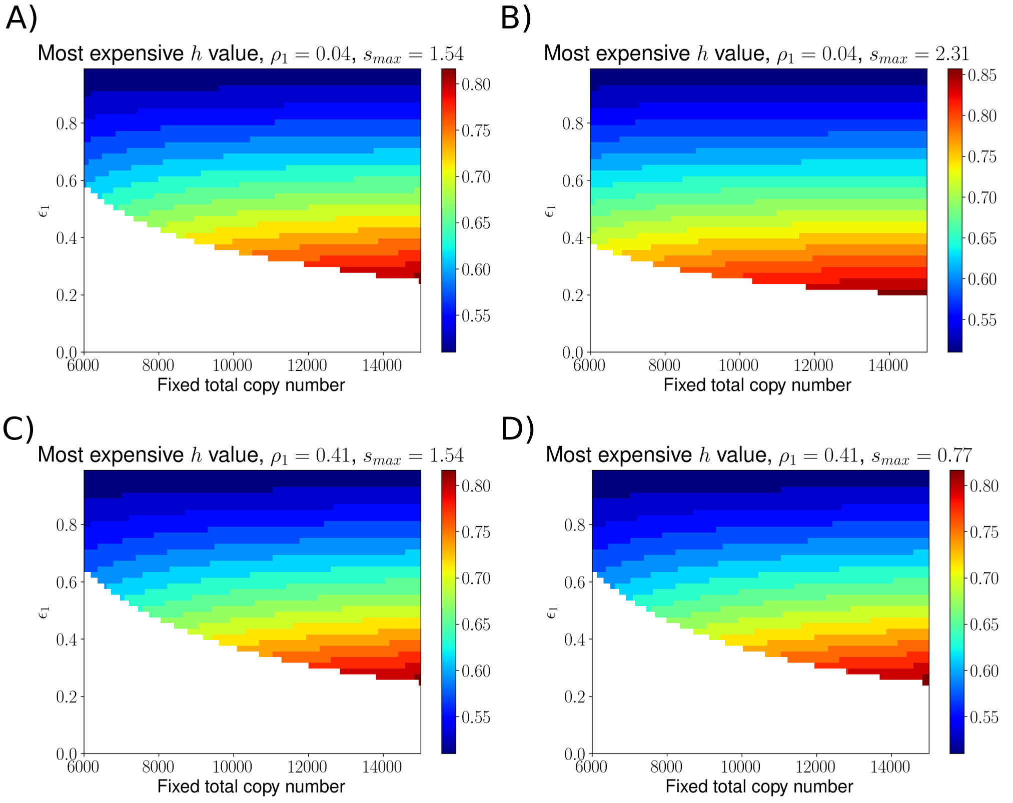

Figure 5: **The existence of intermediate heteroplasmy values is a robust feature of the saturating output model.** We show the value of h_{max}, the most expensive heteroplasmy value at constant copy number, as a function of total copy number and ϵ_1 (describing mutant pathology). White regions correspond to $h_{max} = 1$. **A)** Using our default parameter values, an intermediate h_{max} exists for large enough mutant functionality ϵ_1. **B)** The parameter s_{max} is increased by 50% with minimal effect on the output. We kept the parameter k fixed as it defines the amount of proton leak (the resource consumption rate at zero energy output) which agrees with the amount of proton leak in the linear output model whose parameters are based on experimental data. **C)** The parameter ρ_1 is increased by an order of magnitude with minimal effect on the output. **D)** The parameter ρ_1 is increased by an order of magnitude and s_{max} is decreased by 50%. Again, change in h_{max} are small.

5.3 Comparison of the cost of different control mechanisms

In the main text we introduced four different feedback controls and compared their mean costs. Here, we additionally show the means and variances of the wildtypes, mutants and cost up to ~ 82 years resulting from stochastic simulations. The parameter values we used to compare the four controls are summarised in Table 3 for clarity. We observe that the increase in cost for the linear control with $\delta = 0$ is mainly caused by an increase in mean mutant copy number.

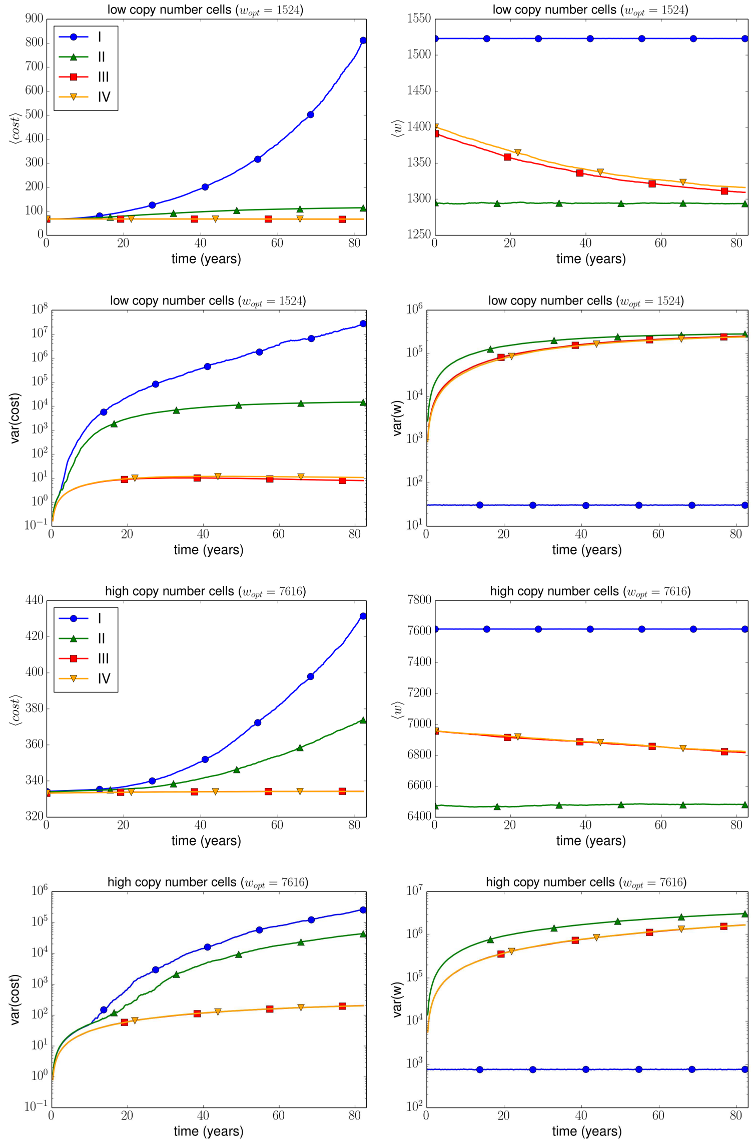

Figure 6: **Wildtype, mutant and cost dynamics for four different control strategies.** Dynamics are shown for the four controls I, II, III, and IV defined in Table 3. Again, we see that the effects of the control are more noticeable in low copy number cells. Parameter are set as given in Table 3. Values for w_{opt} are those for the saturating output model at low and high copy number. The free parameters in control III and IV (δ and η) were optimised over initial conditions in the range $h \in [0, 0.2]$. For the optimization the default cost function parameters were used as well as $\epsilon_1 = 0.3$.

Fig 7 shows the optimal values for δ in the linear feedback control $\lambda(w, m) = \mu + c_1(N_{ss} - (w + \delta m))$ as a function of ϵ_1. Stochastic simulations starting in steady state at either $h_0 = 0.1$ or $h_0 = 0.8$ were performed for $T = 10^4$ days. The mean integrated cost over these 10^4 days was evaluated for different values of δ, and the optimal δ values are shown. This was done for both the linear and the saturating model. The general trend is, as was shown for $T = 100$ in the main text, the lower ϵ_1 the lower the optimal δ.

Label	Control	optimal parameters satisfying our two constraints
I	$\lambda(w) = \mu + c_1(w_{opt} - w)$	$c_1 = \alpha_R \mu / w_{opt}$
II	$\lambda(w+m) = \mu + c_1(w_{opt} - (w+m))$	$c_1 = \alpha_R \mu / w_{opt}$
III	$\lambda(w+\delta m) = \mu + c_1(w_{opt} - (w+\delta m))$	$c_1 = \alpha_R \mu / w_{opt}$<br>$\delta$ is optimised
IV	$\lambda(w,m) = \frac{\mu(\alpha_R(w_{opt}-w-\eta m)+w+\eta m)}{(w+m)}$	$\alpha_R = 10.0$<br>η is optimised

Table 3: **Parameter values for the four different control mechanisms we employ.** Two parameters of each control are set by the two constraints we impose. The parameter α_R was proposed to lie in the range 5-17 [3] and here we used $\alpha_R = 10$. The values for δ and η are found by optimizing our cost function over the steady states corresponding to our initial conditions. We used 50 initial conditions equally spread over the range $h_0 \in [0, 0.2]$. The two values used for w_{opt} are 1524 and 7616 (Table 2). We further use $\mu = 0.07$ day^{-1}.

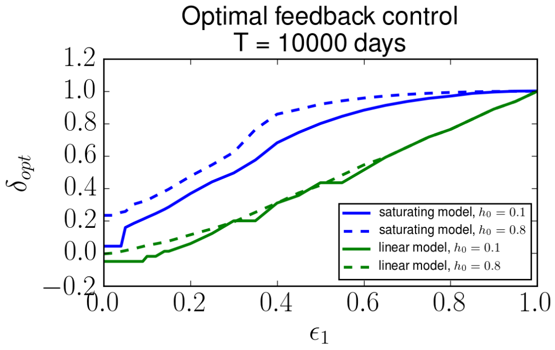

Figure 7: **At long times and high heteroplasmies, energy sensing control becomes suboptimal.** The optimal value of δ in a linear feedback control is shown as a function of ϵ_1. Here we used $T = 10^4$ days (optimization time) and low copy numbers for both the linear and saturating model. The solid and dashed lines correspond to trajectories starting at $h_0 = 0.1$ and $h_0 = 0.8$, respectively. The less resources the mutants consume (and the less output they therefore produce) the lower their optimal contribution to the control.

6 Zinc Finger nuclease treatment model

6.1 Visualisation of Zinc Finger concentrations during treatment

As explained in the main text, we simulate the treatment of cells with mitochondrially targeted zinc finger nucleases (mtZFNs). The concentration of mtZFNs is modelled by Eq. (9) in the main text, which is shown for different treatment durations (different values for b) in Fig 8A.

6.2 Heteroplasmy values can increase after nuclease treatments

As mentioned in the main text, there is a possibility for cellular heteroplasmy to increase after a treatment has been applied. This is true especially if the selectivity of the treatment is low (i.e. ξ is close to 1) and the initial heteroplasmy of a cell is high; in this case treating a cell may even eliminate all wildtype mitochondria, increasing heteroplasmy to 1. To model the extent of this effect we initialise a cell with a given heteroplasmy h_0, and let it undergo one round of treatment and recovery after which the final heteroplasmy is recorded. This process is repeated to obtain the probability that, given an initial heteroplasmy h_0, the final heteroplasmy after treatment exceeds h_0 ($P(h_{\text{final}} > h_0|h_0)$). Figs 8B,C show these probabilities as a function of h_0 and selectivity parameter ξ, for initial mtDNA copy numbers 500 and 5000. We observe that the effect-size is larger for low copy number cells. Fig 8D shows an example of the distribution of post-treatment heteroplasmies. The recovery time used in the simulations is 30 days which is long enough for the cells to recover their initial copy numbers and short enough for the change in h to be almost completely due to treatment, rather than due to naturally occurring random drifts in heteroplasmy values. Chances of increasing heteroplasmy are highest when h_0 is very high or very low (if h_0 is low the low mutant copy numbers increase the effect of stochastic fluctuations). In the examples shown, when $\xi <= 0.6$ (i.e. for every mutant that is cleaved, 0.6 wildtypes are cleaved) increases in heteroplasmy are very unlikely to occur.

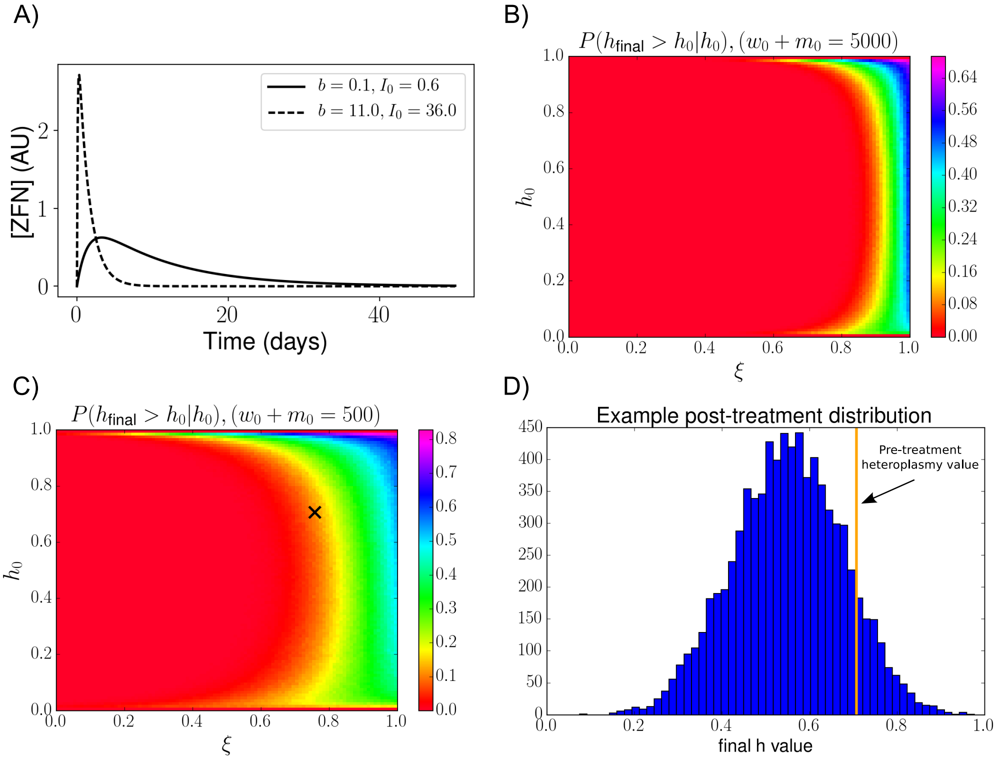

Figure 8: **Zinc Finger Nuclease concentrations for short and long treatments. A)** Here we show the concentration of mitochondrially targeted Zinc Fingers as modelled by Eq. (9) in the main text. The parameter values for the short and strong treatment illustrated here ($I_0 = 36, b = 11$) are similar to those found in fitting the model to the data. For the mtZFN degradation rate we used $\mu_Z = \log(2)$ day^{-1} (corresponding to a mtZFN half-life of 1 day). **There exists a possibility of increasing heteroplasmy levels through treatment. B)** The probability of increasing heteroplasmy above its initial pre-treatment value h_0, after one round of treatment and recovery, is shown as a function of h_0 and ξ. Cells are initialised with a total copy number of 500. The cross indicates the parameters used in figure (D). The parameter values for I_0, b and c_1 are fixed at: $(I_0, b, c_1) \approx (39, 20, 3 \times 10^{-4})$; these values provide good fits to experimental data when assuming a total initial copy number of 500. We used $\delta = 1$. **C)** Similar to figure (B), but now cells are initialised with a total copy number of 5000; in these large copy number cells stochastic fluctuations in copy number have less effect and the probabilities of exceeding initial heteroplasmy values are smaller compared to figure (B). **D)** An example of a distribution of post-treatment heteroplasmy values is shown using parameters h_0 and ξ as indicated by the cross in figure (B). The orange line indicates the value of h_0 (the heteroplasmy that was present before the treatment started).

6.3 Measurement of mtZFN expression profile

It was previously shown that the mtZFN pairing NARPd(+) and COMPa(-) specifically cleaves mtDNA with the m.8993T>G mutation [29, 30, 31]. We confirmed the transient expression profile of mtZFNs used in the modelling by transfecting 143B cells with a plasmid encoding NARPd(+) that co-expresses fluorescent marker protein mCherry. At 24 hours post-transfection, transfected cells were sorted using fluorescence activated cell sorting (FACS), to ensure a homogenously transfected sample (Fig 9A). Cells were harvested at 24 hours, and the remainder returned to culture dishes, to be harvested at later time points. Total protein was extracted from cells and analysed by western blotting, using antibodies to the HA epitope. Coomassie (CM) staining was used to verify equal loading of the gel. For full protocol see Ref. [32]. We observe that expression of mtZFNs is almost undetectable by 96 hours, and is totally undetectable by 120 hours (Fig 9B).

6.4 MtDNA copy number measurements in heteroplasmic cells

To determine the absolute mtDNA copy number in the pre-treatment 80% heteroplasmy cells, a plasmid construct containing the mitochondrial region of interest was created. The exact molecular weight of the dsDNA plasmid molecule was calculated based on nucleotide composition (3318.8 kDa). The plasmid and experimental sample concentrations were measured in triplicate with a Qubit fluorometer using the Qubit dsDNA BR Assay Kit (ThermoFisher Scientific).

Three 10-fold plasmid serial dilutions were made (spanning 8 orders of magnitude) and analysed by quantitative PCR. Five similar dilutions of the experimental sample were measured in triplicate. Highest and lowest values were removed from further analysis to reduce the effect of pipetting errors. The calculated mtDNA copy number per μL sample (1.13×10^7) was determined as the average number for

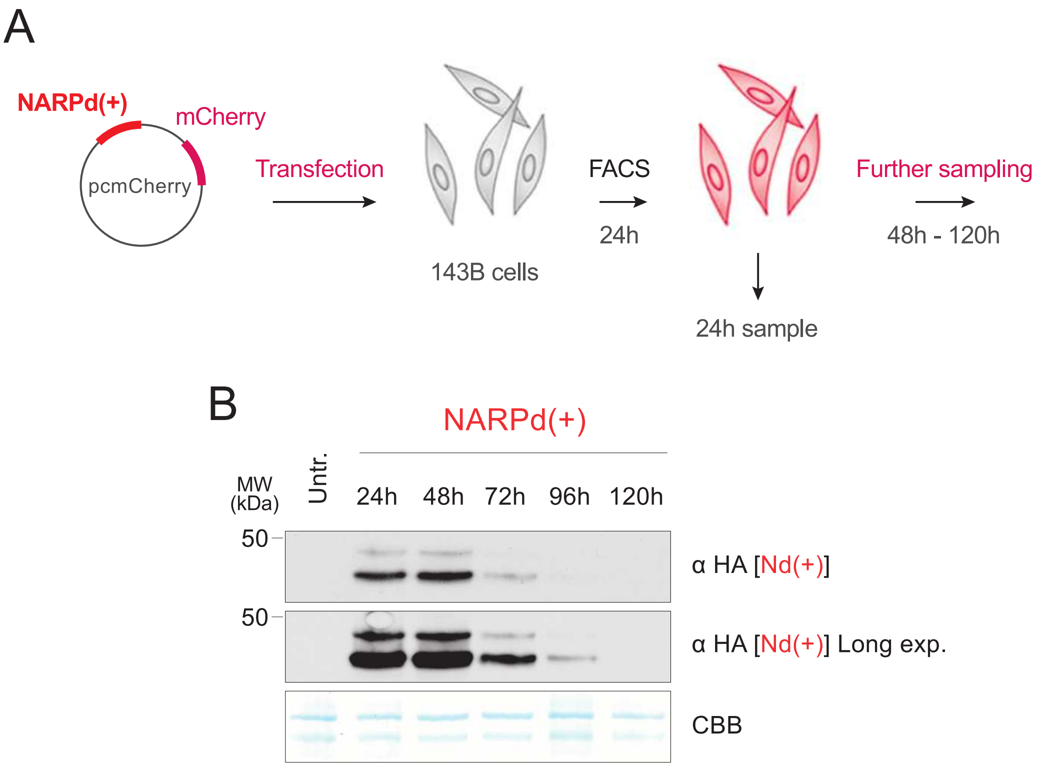

Figure 9: **mtZFN expression profile during transient transfection of 143B cells. A)** Here we show a schematic of the experiments involving i) transient transfection of high-heteroplasmy cells with plasmids expressing mtZFN monomers and fluorescent marker proteins, ii) FACS-based selection of cells expressing both mtZFN monomers (NARPd(+) and COMPa(-)), and iii) phenotypic evaluation of treated cells. Technical details are provided in Ref. [32]. **B)** Western blots showing the mtZFN expression profile indicate that the mtZFNs are almost undetectable at 96 hours post-transfection, and completely undetectable at 120 hours. Details of the protocol are provided in Ref. [32].

each unknown replicate using the average result based on each of the three standard curves. The number of cells per μL was calculated based on the mass of DNA per single diploid cell (6.57 pg) [33]. As 143B cells are mildly aneuploid, this is likely to be a small underestimate.

Primers and probe used for qPCR:
mtDNA 3211 F: 5 CACCCAAGAACAGGGTTTGT 3,
mtDNA 3298 R: 5 TGGCCATGGGTATGTTGTTAA 3
mtDNA 3242 probe: 6-Fam-TTACCGGGCTCTGCCATCT-Tamra

Using this method, we calculate that each pre-treatment 143B cell (with heteroplasmy 0.8) in the initial cycle of iterative mtZFN treatments contained 889 ± 214 (S.E.) mtDNA copies per cell. Based on these measurements, we use a total initial copy number of 900 in our MCMC simulations used to fit the experimental data provided in Ref. [31].

6.5 Bayesian inference model outputs

Fig 10 shows posterior distributions and inference traces following our Metropolis sampling procedure. Some of the distributions shown are log-transforms of the actual parameters in our model. The posterior distribution of the parameter b reaches the upper bound set by the prior (specified below). This is due to a degeneracy in the model for large values of b because, in this case, the mtZFN concentration can be written as

$$[ZFN](t) = \frac{I_0}{b} e^{-\mu_z t}. \tag{22}$$

Therefore, as long as the ratio I_0/b is constant, a larger value for b does not influence the mtZFN dynamics and therefore the mtDNA dynamics. Because it is the ratio I_0/b that determines the mtZFN dynamics at large b, we have performed the inference using this ratio rather than the parameter I_0 itself.

Fig 11 shows that our model predictions of mtZFN expression profiles are in broad accord with the experimental data obtained in this study. Our prior distribution on the treatment duration parameter b allows for much more slowly decaying mtZFN concentrations, but our inference selected values for b that predict very low mtZFN concentrations 5 days post-transfection, agreeing with the experimental data.

Prior distributions used are: δ: $\mathcal{N}(1.0, 1.0)$ bounded between 0 and 10; $\log_{10} I_0/b$: $U(-1, 3)$; $\log_{10} b$: $U(-3, 3)$; ξ: $U(0, 1)$; $\log_{10} c_1$: $U(-7, -2)$; σ_h: $U(0, 0.5)$; σ_T: $U(0, 0.5)$. The priors of the standard

deviations were chosen to be $U(0, 0.5)$ because both h and T mostly lie in the range $[0, 1]$ and experimental data indicate a standard deviation smaller than 0.5. The prior distribution of c_1 was motivated by investigating the general behaviour of mtDNA dynamics for various values of c_1. We found that if $c_1 = 10^{-2}$ the control is unrealistically strong whereas if $c_1 = 10^{-7}$ it takes unrealistically long time to return to a steady state value when out of equilibrium.

6.6 Parameter values used in Fig 6 in the main text

Figs 6A,B in the main text were obtained using stochastic Gillespie simulations in which mtDNA dynamics were simulated using Eq. (12) in the main text. 10^4 cells were simulated for 400 days with the following initial heteroplasmy distributions, all with mean $\langle h \rangle = 0.8$: i) all h values fixed at 0.8 (left), ii) Beta$(12, 3)$ (middle), and iii) Beta$(0.4, 0.1)$ (right). The following parameters were used: $w_{opt} = 5000$, $I_0 = 39.6$, $b = 12.4$, $\xi = 0.76$, $c_1 = 5.1 \times 10^{-5}$, $\delta = 1.0$ and $\mu = 0.07$ day^{-1}. These parameter values were chosen because they provide good fits to the experimental data in Ref. [31].

Fig 6C is obtained from stochastic simulations using our default cost function parameters for 'low' copy number settings (Table 2), and using the following mtZFN treatment parameters (which provided a good fit to experimental data): $b = 11.9, \xi = 0.76, c_1 = 2.5 \times 10^{-4}, \delta = 1.0$ and $\mu = 0.07$ day^{-1}. For each value of ϵ_1, the value for I_0 corresponding to the minimum cost over a simulation time of 400 days was found.

The short and strong treatment in Fig 6D (blue line) uses $b = 11.9, \xi = 0.76, c_1 = 2.5 \times 10^{-4}, \delta = 1.0$, and $\mu = 0.07$ day^{-1}. We further used $I_0 = 47.1$ which was found to be optimal for $\epsilon_1 = 0.2$ (the cost function corresponding to this value of ϵ_1 is shown in the figure). The long and weak treatment uses $b = 0.1$ and $I_0 = 0.6$ (the optimal treatment strength when using $\epsilon_1 = 0.2$ and $b = 0.1$). For the more selective treatment (magenta line) we set $\xi = 0.4$, $b = 11.9$ and $I_0 = 41.75$ (the optimal treatment strength under these ξ and b). The heteroplasmy mappings corresponding to short and long treatments shown in Fig 6E were obtained from stochastic simulations using parameters identical to the ones for the short and long treatments in Fig 6D. We note that in finding $I_{0,opt}$, we initialised all cells at identical states. When a distributions of initial states is used, the variance that is now present is likely to affect the optimal treatment strength.

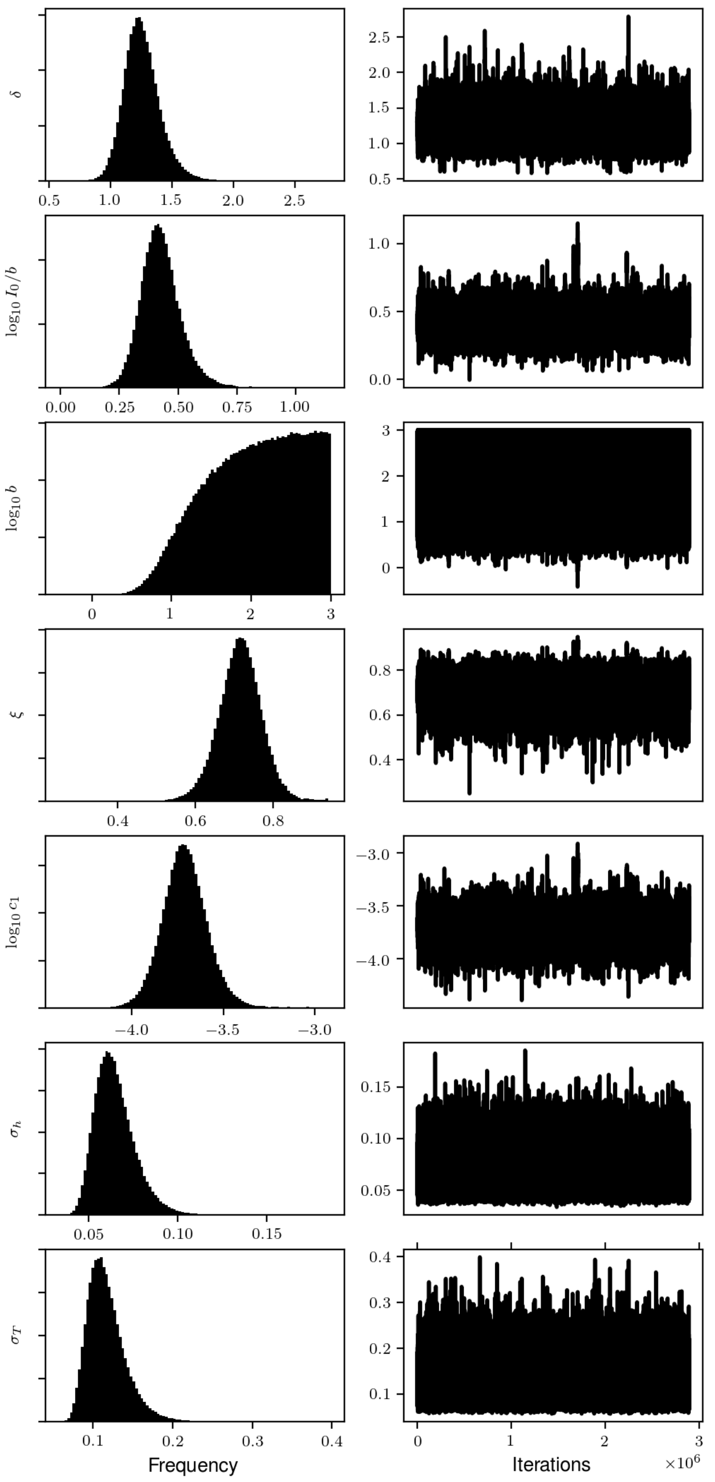

Figure 10: **Posterior mtZFN treatment parameter distributions.** Here we show our posterior distributions obtained after running our MCMC algorithm (left) as well as the corresponding sample values (right). Prior distributions are provided in the text. The posterior of $\log_{10} b$ is cut off due to a degeneracy in our model (Eq. (22)), which does not affect our model predictions.

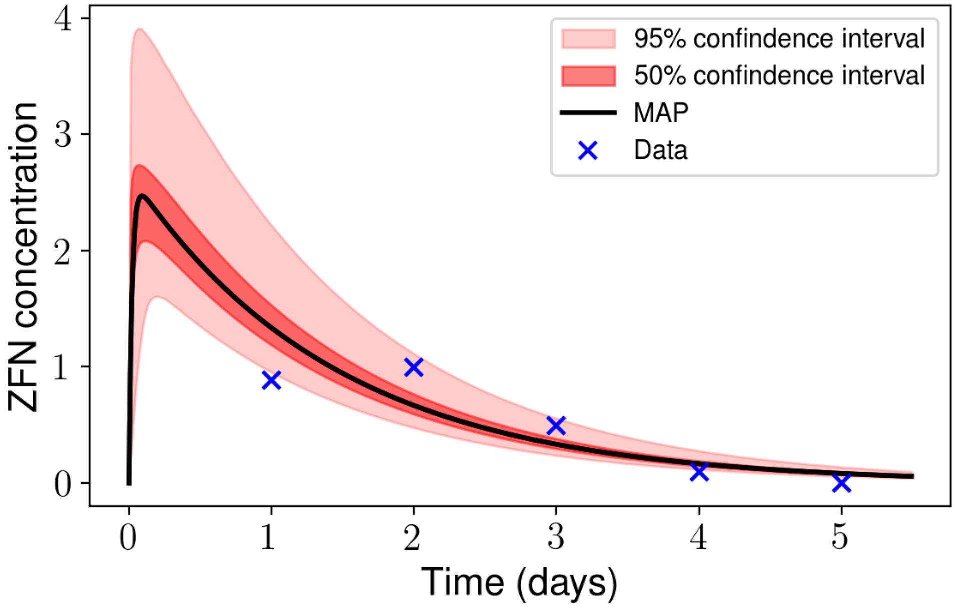

Figure 11: **Predictions of mtZFN expression are broadly consonant with experimental data.** Drawing from our posterior distributions for I_0 and b obtained through Metropolis sampling, we show 50% and 95% confidence intervals of our predicted mtZFN expression profile (solid black line denotes the maximum a posteriori (MAP) estimate). Data points are obtained through quantification of the western blots shown in Fig 9B and were subsequently rescaled to investigate whether our model can broadly account for the experimentally observed dynamics (our predicted mtZFN concentrations are proportional to the measurement data points with an arbitrary proportionality constant).

References

[1] Van Kampen, N. G. *Stochastic processes in physics and chemistry*, volume 1. Elsevier, (1992).

[2] Johnston, I. G. and Jones, N. S. Evolution of cell-to-cell variability in stochastic, controlled, heteroplasmic mtdna populations. *The American Journal of Human Genetics* **99**(5), 1150–1162 (2016).

[3] Chinnery, P. F. and Samuels, D. C. Relaxed replication of mtDNA: a model with implications for the expression of disease. *The American Journal of Human Genetics* **64**(4), 1158–1165 (1999).

[4] Capps, G. J., Samuels, D. C., and Chinnery, P. F. A model of the nuclear control of mitochondrial DNA replication. *Journal of theoretical biology* **221**(4), 565–583 (2003).

[5] Monternier, P.-A., Marmillot, V., Rouanet, J.-L., and Roussel, D. Mitochondrial phenotypic flexibility enhances energy savings during winter fast in king penguin chicks. *Journal of Experimental Biology* **217**(15), 2691–2697 (2014).

[6] Salin, K., Roussel, D., Rey, B., and Voituron, Y. David and Goliath: a mitochondrial coupling problem? *Journal of Experimental Zoology Part A: Ecological Genetics and Physiology* **317**(5), 283–293 (2012).

[7] Brand, M. D., Harper, M.-E., and Taylor, H. C. Control of the effective P/O ratio of oxidative phosphorylation in liver mitochondria and hepatocytes. *Biochemical Journal* **291**(3), 739–748 (1993).

[8] Yadava, N. and Nicholls, D. G. Spare respiratory capacity rather than oxidative stress regulates glutamate excitotoxicity after partial respiratory inhibition of mitochondrial complex i with rotenone. *The Journal of neuroscience* **27**(27), 7310–7317 (2007).

[9] Hill, B. G., Dranka, B. P., Zou, L., Chatham, J. C., and Darley-Usmar, V. M. Importance of the bioenergetic reserve capacity in response to cardiomyocyte stress induced by 4-hydroxynonenal. *Biochemical Journal* **424**(1), 99–107 (2009).

[10] Pfleger, J., He, M., and Abdellatif, M. Mitochondrial complex ii is a source of the reserve respiratory capacity that is regulated by metabolic sensors and promotes cell survival. *Cell death & disease* **6**(7), e1835 (2015).

[11] Johnston, I. G., Rickett, B. C., and Jones, N. S. Explicit tracking of uncertainty increases the power of quantitative rule-of-thumb reasoning in cell biology. *Biophysical journal* **107**(11), 2612–2617 (2014).

[12] Jajoo, R., Jung, Y., Huh, D., Viana, M. P., Rafelski, S. M., Springer, M., and Paulsson, J. Accurate concentration control of mitochondria and nucleoids. *Science* **351**(6269), 169–172 (2016).

[13] Hinkle, P. C. P/O ratios of mitochondrial oxidative phosphorylation. *Biochimica et Biophysica Acta (BBA)-Bioenergetics* **1706**(1), 1–11 (2005).

[14] Lee, C., Gu, Q., Xiong, Y., Mitchell, R., and Ernster, L. P/O ratios reassessed: mitochondrial P/O ratios consistently exceed 1.5 with succinate and 2.5 with nad-linked substrates. *The FASEB Journal* **10**(2), 345–350 (1996).

[15] Sriskanthadevan, S., Jeyaraju, D. V., Chung, T. E., Prabha, S., Xu, W., Skrtic, M., Jhas, B., Hurren, R., Gronda, M., Wang, X., et al. Aml cells have low spare reserve capacity in their respiratory chain that renders them susceptible to oxidative metabolic stress. *Blood* **125**(13), 2120–2130 (2015).

[16] Nicholls, D. G. Mitochondrial ion circuits. *Essays in biochemistry* **47**, 25–35 (2010).

[17] Jin, K., Musso, G., Vlasblom, J., Jessulat, M., Deineko, V., Negroni, J., Mosca, R., Malty, R., Nguyen-Tran, D.-H., Aoki, H., et al. Yeast mitochondrial protein–protein interactions reveal diverse complexes and disease-relevant functional relationships. *Journal of proteome research* **14**(2), 1220–1237 (2015).

[18] Cherry, J. M., Hong, E. L., Amundsen, C., Balakrishnan, R., Binkley, G., Chan, E. T., Christie, K. R., Costanzo, M. C., Dwight, S. S., Engel, S. R., et al. Saccharomyces genome database: the genomics resource of budding yeast. *Nucleic acids research* , D700–D705 (2011).

[19] Phillips, R., Kondev, J., Theriot, J., and Garcia, H. *Physical biology of the cell*. Garland Science, (2012).

[20] Herrmann, J. M., Woellhaf, M. W., and Bonnefoy, N. Control of protein synthesis in yeast mitochondria: the concept of translational activators. *Biochimica et Biophysica Acta (BBA)-Molecular Cell Research* **1833**(2), 286–294 (2013).

[21] Osman, C., Noriega, T. R., Okreglak, V., Fung, J. C., and Walter, P. Integrity of the yeast mitochondrial genome, but not its distribution and inheritance, relies on mitochondrial fission and fusion. *Proceedings of the National Academy of Sciences* **112**(9), E947–E956 (2015).

[22] Myers, M. W. and Bosmann, H. B. Mitochondrial protein content and enzyme activity of reuber hepatoma h-35. *Cancer research* **34**(8), 1989–1994 (1974).

[23] Brandt, F., Etchells, S. A., Ortiz, J. O., Elcock, A. H., Hartl, F. U., and Baumeister, W. The native 3d organization of bacterial polysomes. *cell* **136**(2), 261–271 (2009).

[24] Dill, K. A., Ghosh, K., and Schmit, J. D. Physical limits of cells and proteomes. *Proceedings of the National Academy of Sciences* **108**(44), 17876–17882 (2011).

[25] Rafelski, S. M., Viana, M. P., Zhang, Y., Chan, Y.-H. M., Thorn, K. S., Yam, P., Fung, J. C., Li, H., Costa, L. d. F., and Marshall, W. F. Mitochondrial network size scaling in budding yeast. *Science* **338**(6108), 822–824 (2012).

[26] Lane, N. and Martin, W. F. Mitochondria, complexity, and evolutionary deficit spending. *Proceedings of the National Academy of Sciences* **113**(6), E666 (2016).

[27] Flamholz, A., Phillips, R., and Milo, R. The quantified cell. *Molecular biology of the cell* **25**(22), 3497–3500 (2014).

[28] Shmookler, R. R. and Goldstein, S. Mitochondrial DNA in mortal and immortal human cells. genome number, integrity, and methylation. *The Journal of biological chemistry* **258**(15), 9078–9085 (1983).

[29] Minczuk, M., Papworth, M. A., Miller, J. C., Murphy, M. P., and Klug, A. Development of a single-chain, quasi-dimeric zinc-finger nuclease for the selective degradation of mutated human mitochondrial DNA. *Nucleic acids research* **36**(12), 3926–3938 (2008).

[30] Gammage, P. A., Rorbach, J., Vincent, A. I., Rebar, E. J., and Minczuk, M. Mitochondrially targeted ZFNs for selective degradation of pathogenic mitochondrial genomes bearing large-scale deletions or point mutations. *EMBO molecular medicine* **6**(4), 458–466 (2014).

[31] Gammage, P. A., Gaude, E., Van Haute, L., Rebelo-Guiomar, P., Jackson, C. B., Rorbach, J., Pekalski, M. L., Robinson, A. J., Charpentier, M., Concordet, J.-P., et al. Near-complete elimination of mutant mtDNA by iterative or dynamic dose-controlled treatment with mtZFNs. *Nucleic Acids Research* , 7804–7816 (2016).

[32] Gammage, P. A., Van Haute, L., and Minczuk, M. Engineered mtZFNs for manipulation of human mitochondrial DNA heteroplasmy. *Mitochondrial DNA: Methods and Protocols* , 145–162 (2016).

[33] Morton, N. E. Parameters of the human genome. *Proceedings of the National Academy of Sciences* **88**(17), 7474–7476 (1991).
